# UROREC: A cystometry and electromyography recording system for analyzing urinary incontinence parameters in rat models

**DOI:** 10.1371/journal.pone.0356999

**Published:** 2026-08-26

**Authors:** Ramon Eduardo Cortina, Luis Padilla, Pilar Hazel Carranza-Castro, Fernando Pérez Escamirosa, Daniel Lorias Espinoza, Arturo Minor Martínez

**Affiliations:** 1 Department of Electrical Engineering, Bioelectronics Section, Center for Research and Advanced Studies, Mexico City, Mexico; 2 Experimental Surgery Service, Microsurgery Unit, Centro Médico Nacional 20 de Noviembre ISSSTE, Mexico City, Mexico; 3 Instituto de Ciencias Aplicadas y Tecnología (ICAT), Universidad Nacional Autónoma de México (UNAM), Ciudad de México, México; Weill Cornell Medical College, UNITED STATES OF AMERICA

## Abstract

Preclinical research on the lower urinary tract relies heavily on murine models, which necessitates specialized equipment for acquiring concurrent electromyography and cystometry. However, commercial systems are often expensive and bulky, requiring dedicated workstations. These limitations hinder the reproducibility of complex urodynamic studies and restrict experimental accessibility. To address these limitations, we present the UROREC system, which is an accessible, compact, and reliable data acquisition platform designed to simultaneously record electromyography of the external urethral sphincter and intravesical pressure. We established the system’s robustness through benchtop electrical characterization and validated it via in vivo proof-of-concept pilot experiments in urethane-anesthetized female Wistar rats. Electrical testing confirmed a common-mode rejection ratio of 98.4 dB, an input-referred noise of 1.53 µV RMS, and linearity of R^2^ = 0.9999. Meanwhile, the pressure sensor exhibited a maximum hysteresis of 0.29% at full scale. In vivo, the device reliably digitized electromyography signals at 20 ksps and pressure signals at 100 sps in both a healthy physiological state and an acetic acid–induced overactive bladder model. UROREC precisely quantified 18 cystometric and electromyographic parameters consistent with the existing literature. The robustness of these in vivo recordings was confirmed by quantifying the signal-to-noise ratio, demonstrating high operational reliability and data integrity across all experimental sessions. Furthermore, the system accurately captured high sensitivity to specific pathophysiological shifts induced by acid instillation, with a significant reduction in both the inter-contraction interval (*p* < 0.001) and tonic activity duration (*p* < 0.001). These results validate UROREC as a robust, cost-effective alternative that makes high-fidelity urodynamic monitoring more accessible and promotes methodological reproducibility in lower urinary tract research.

## Introduction

The study of lower urinary tract (LUT) dysfunction resulting from neurological diseases or injury is primarily conducted experimentally in animal models [1]. This research requires access to workstations, surgical equipment, specialized accessories, and data acquisition devices to generate accurate records for subsequent analysis [2]. These devices have contributed to the development and study of new therapies and surgical methods to address conditions such as organ prolapse, pelvic floor dysfunction, and overactive bladder (OAB) [3]. These conditions often result in urinary incontinence (UI), which is defined as the involuntary loss of urine [4]. UI associated with OAB affects a wide demographic spectrum, ranging from children and adolescents to adults and elderly individuals. Studies have projected that approximately 423 million people would be affected by some form of UI by 2018 [5]. However, recent estimates for 2024 have indicated a substantial increase, suggesting that between 700 million and 1.2 billion individuals residing in major urban centers may be affected by OAB-related UI [6]. These discrepancies underscore the difficulty of obtaining definitive global statistics and highlight the growing magnitude of this public health problem.

Accurate diagnosis and treatment planning for UI require imaging studies or urodynamic testing [7]. Conventional management strategies encompass a wide spectrum of approaches, ranging from pharmacotherapy to surgical interventions targeting the underlying etiology [8]. However, some patients do not respond to conventional therapies [9], making it necessary to research and develop new therapeutic alternatives, which were initially validated in animal models such as rats. To evaluate the efficacy of new treatments and their interaction with the underlying pathology, researchers often rely on *ex vivo* or *in vivo* studies, with the latter being the most prevalent [10]. A primary approach involves the use of laboratory animal models to replicate specific pathological conditions, such as denervation or nerve transection [11,12], spinal cord injury [13], or OAB [14,15]. These models facilitate the study of disease progression, tissue damage, and the onset of UI. Monitoring parameters such as filling pressure, bladder capacity, electromyographic (EMG) signals, and micturition episode duration is essential for evaluating experimental treatments [16].

However, these studies require specialized hardware for biosignals acquisition, including low-noise amplifiers, isolated power supplies, and dedicated computer workstations [17]. Moreover, strict adherence to approved bioethics protocols, the availability of *in vivo* specimens, and the acquisition of surgical equipment, anesthetic agents, and specialized technical support significantly increase operating costs [18]. These logistical and financial barriers impede the feasibility of such experimental procedures and create a substantial disparity between well-funded laboratories and smaller research groups that lack the equipment necessary to replicate the reported results. Although commercial systems from companies such as Stanford Research Systems, Grass Instruments, and Astro-Med allow for *in vivo* experimentation in various animal models, they often rely on specialized workstations, bulky hardware, or high acquisition costs, which restrict data collection to designated areas of the laboratory.

This study addresses logistical and financial limitations while promoting methodological reproducibility by presenting the development and rigorous in vivo pilot validation of UROREC, an accessible, compact, and highly reliable data acquisition platform for synchronous cystometry (CMG) and external urethral sphincter electromyography (EUS-EMG). This reproducible system is designed for seamless integration into standard murine urodynamic protocols [10,19], eliminating dependence on dedicated and restrictive commercial workstations. This work primarily aims to demonstrate the analytical robustness of UROREC in acquiring, processing, and logging concurrent CMG and EUS-EMG activity, rather than to explore novel or complex biological mechanisms. Furthermore, the system’s efficacy is validated by quantifying established baseline physiological benchmarks and confirming its ability to detect pathophysiological alterations in an OAB model. UROREC provides a scalable framework that makes physiological research more accessible, enabling laboratories to conduct complex LUT studies without compromising data integrity.

## Materials and methods

**Fig 1:**
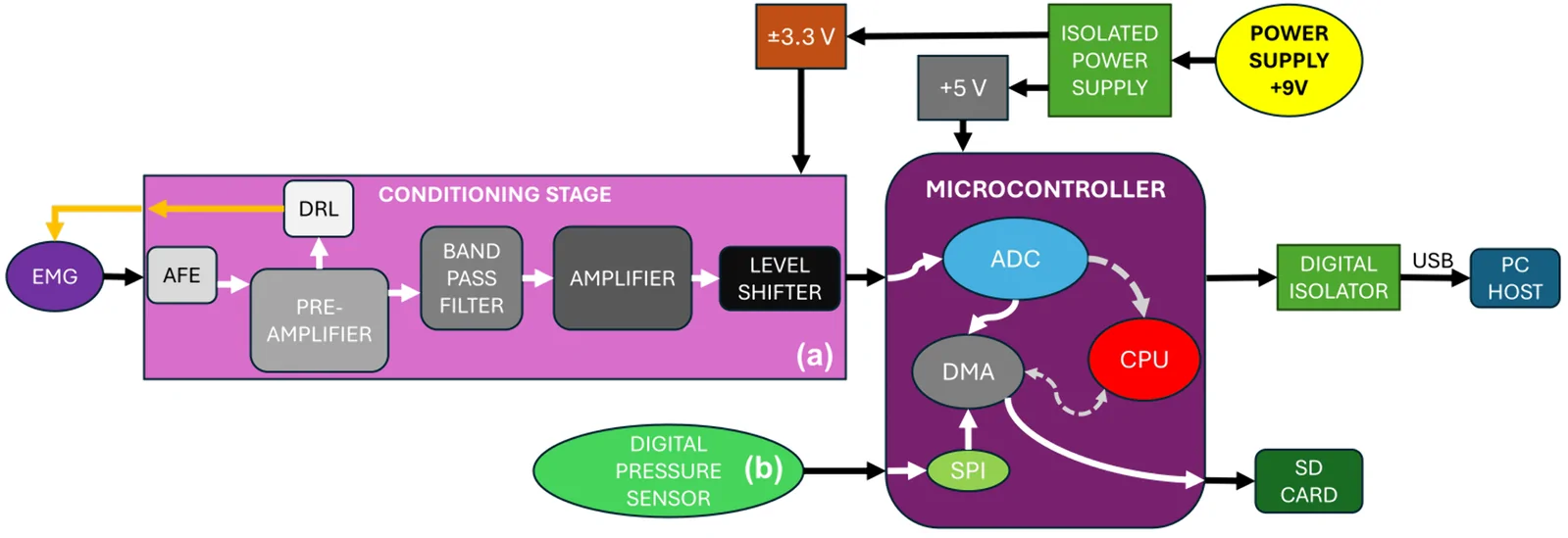
Block diagram that illustrates the integration of the analog conditioning stage. (a) The EMG, (b) the pressure interface, and (c) the digital processing and interface unit. The arrows show the data flow and electrical isolation used to ensure signal integrity and safety during the experiments.

The UROREC is a custom-designed device that simultaneously records EUS-EMG through implanted electrodes and CMG through a catheter. Current protocols reported in the literature typically employ data acquisition systems with sampling frequencies of 10,000–25,000 samples per second (sps) [15,20]. To meet these standards, the device was designed and configured to achieve a maximum sampling rate of 20 ksps, a specification validated experimentally [21]. The UROREC architecture consists of three main modules: a) the EMG acquisition circuit; b) the cystometry acquisition circuit; and c) the digital processing and interface unit. Fig 1 shows the block diagram of the UROREC system.

### Electromyography acquisition circuit

The acquisition of EMG signals requires a dedicated preamplification stage. This stage uses instrumentation amplifiers configured in differential mode, followed by signal filtering and amplification before digitization with an analog-to-digital converter (ADC). The design uses surface-mounted devices (SMDs) to ensure a compact size. This conditioning stage also integrates a Driven Right Leg (DRL) circuit, which serves as an active feedback mechanism to dynamically minimize common-mode power-line interference and environmental noise before amplification. Detailed components, electronic schematic diagrams, and specific characterization protocols are provided in S1 File. S1 Appendix, Note 1.

### Cystometry acquisition circuit

**Fig 2:**
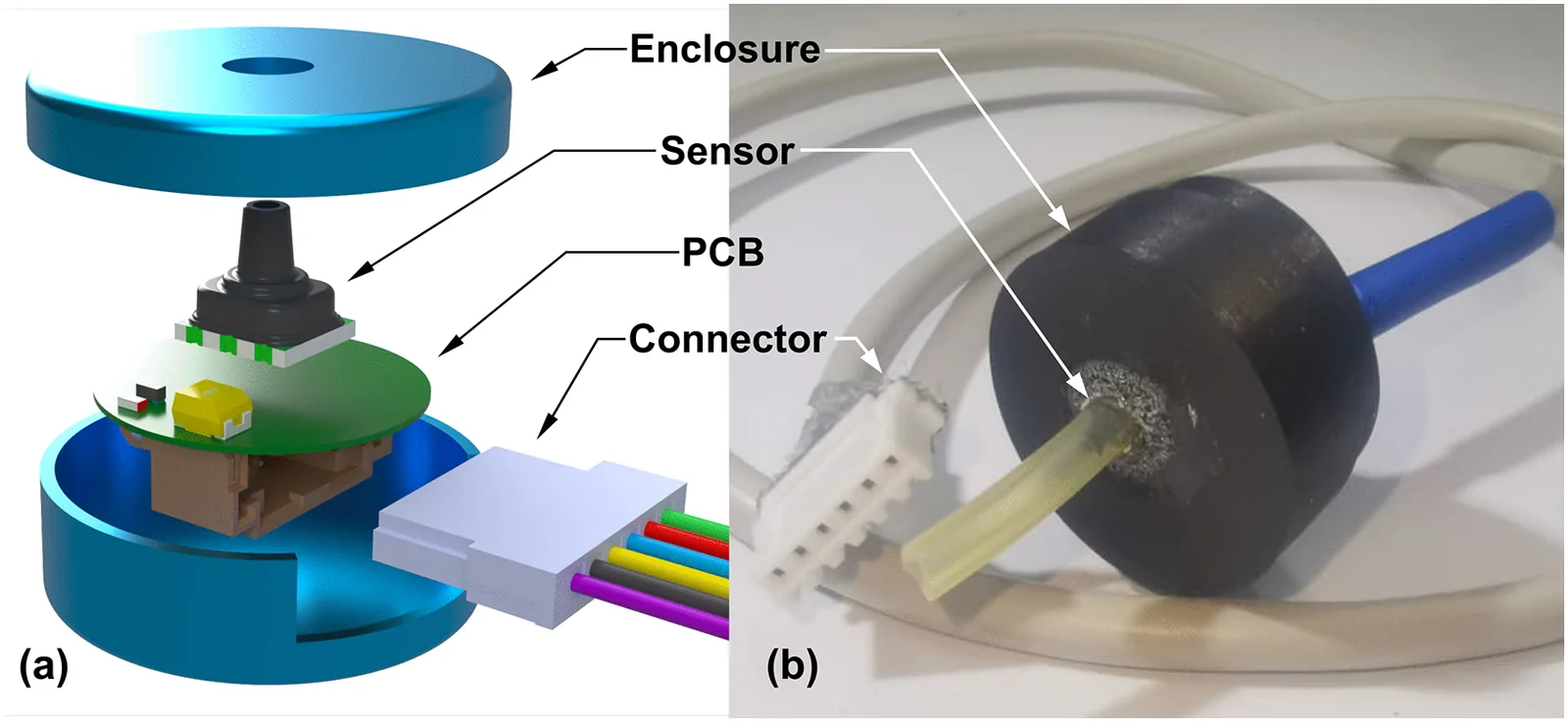
Pressure acquisition module assembly. (a) Internal view showing the custom-designed PCB with the Honeywell ABP2 (ABP2LANT015PGSA3XX) digital sensor and connector. (b) External view showing the 3D-printed PLA casing with the catheter interface. The modular design facilitates sensor placement and replacement.

For the experimental CMG procedures, female Wistar rats were used. These rats exhibit an intravesical pressure range of 30–60 cmH_2_O during micturition, according to studies by [12,21–23]. However, this range can occasionally be exceeded. Based on these physiological parameters, we selected a piezoresistive manometric pressure sensor from the ABP2 series (Honeywell), which is compatible with liquid media owing to its internal silicone gel insulation. This sensor features factory calibration and temperature compensation. Its availability, small size, and competitive price make it easy to integrate into the system. The assembly sensor is presented in Fig 2, and the pressure unit conversion and calibration protocol used is detailed in S1 File. S1 Appendix, Note 3.

### Digital processing and interface unit

The Teensy 4.1 (PJRC) development board serves as the central signal processing unit. It features a 600 MHz ARM Cortex-M7 microcontroller (NXP iMXRT1062) and an internal 12-bit ADC. To ensure galvanic isolation and minimize noise coupling, the power input was processed by a DC–DC converter, which generated an isolated, bipolar, ± 5 V source. The proposed device uses the Direct Memory Access (DMA) controller to manage the simultaneous acquisition of both EMG and CMG data. The DMA controller acts as dedicated hardware that facilitates direct data transfer to an external memory card without CPU intervention, preventing data loss during logging operations.

A computer running a Python program managed real-time data visualization by processing data transmitted via a USB serial connection. The assembly main board and technical specification of data storage are detailed in S1 File. S1 Appendix, Note 4.

### Experimental validation protocol

The validation protocol focused on quantifying the device’s ability to accurately record the established physiological patterns of CMG and EUS-EMG in a murine model. The subjects were evaluated under two conditions: a healthy state and an OAB pathological state induced by acetic acid. This methodology has previously been used to differentiate between physiological states in device validation studies [24,25]. Specifically, the validation protocol quantified the tonic activity of the EUS during the filling phase and the occurrence of phasic bursts. Additionally, changes in bladder pressure before and during micturition were measured. These changes verify the synchronization of EMG events with intravesical pressure variations, as these variations are characteristic of the different phases of the micturition cycle [22].

### Ethical statement

The present study comprised fifteen female Wistar rats, weighing between 250 and 300 grams. The procedures were approved by the Institutional Animal Care and Use Committee (IACUC) at Cinvestav with protocol number E-0040-24, and the study adhered to the ARRIVE 2.0 guidelines for reporting animal research. The animals were housed in Super Rat 1400 cages (Lab Products, LLC) under a 12:12-hour light/dark cycle and had *ad libitum* access to food and water. Three hours prior to each experimental procedure, the rats were separated from their companions and placed in individual cages where trained personnel monitored them at 30-minute intervals for signs of distress.

As this was an acute, non-survival experiment, the humane endpoint was defined as the completion of the urodynamic recording session or any evidence of physiological instability that could not be managed by adjusting the anesthetic dosage. The selection of urethane as the anesthetic agent for the recording phase was made based on its capacity to preserve stable micturition reflexes over extended periods. However, it should be noted that the pharmacological profile of urethane strictly limits its use to terminal procedures [26]. Death was not a planned experimental endpoint. The total duration of the experiment per subject was approximately four hours, including 60 minutes for surgical preparation and electrode implantation, followed by 50 minutes for stabilization and 120 minutes of data recording. Throughout the surgical and recording stages, signs of distress, including but not limited to abnormal posture, piloerection, or respiratory failure, were continuously monitored to ensure timely intervention. However, no animals reached the established humane endpoints prior to the scheduled termination. Following the conclusion of the experiments, all subjects were humanely euthanized with an overdose of isoflurane.

### Animal model of overactive bladder

To induce detrusor hyperactivity, a 0.5% acetic acid solution diluted in physiological saline was prepared in a sterilized glass container one hour before the procedures began. An OAB model can be established by continuously perfusing an acidic solution intravesically through a catheter [14,27]. This process irritates the bladder mucosa and stimulates the afferent C fibers of the urothelium. The result is increased bladder contraction and decreased bladder capacity. This model enables the evaluation of pharmacological agents, alternative treatments such as neuromodulation, and experimental procedures for treating OAB in animal studies [10,15,21].

### Surgical procedures

All surgical procedures were performed under general anesthesia. Isoflurane was administered at a concentration of 5% for induction and 3% for maintenance. It was delivered through a constant flow of pure oxygen. A Surgi-Suite heating pad (Kent Scientific Corporation) was used to maintain the subject’s body temperature throughout the procedure. The specimen was placed in the supine position and secured to the operating table with cotton straps around the upper and lower extremities; only the necessary amount of traction was applied. This immobilization ensured stable recordings by preventing motion artifacts caused by involuntary spasms or tissue manipulation.

An incision was made along the lower midline of the abdomen to expose the bladder and proximal urethra. The skin and muscle layers were dissected via a blunt technique to minimize tissue trauma and prevent bleeding. The tissues were kept hydrated with a physiological saline solution during the procedure. After accessing the abdominal cavity, the EUS muscle was located using the base of the bladder as an anatomical reference. The periurethral adipose and connective tissue were carefully removed to expose the EUS fibers while preserving the adjacent vasculature and nerves. After the EUS was exposed, recording electrodes were implanted to quantify electrical activity before and during micturition. For this study, osteotomy of the pubic symphysis was unnecessary to access the EUS muscle.

### Electrode implantation in the EUS

To record EMG activity, we manufactured custom monopolar electrodes using 50 µm-diameter, 316-stainless-steel wire coated with perfluoroalkoxy alkanes (PFA) (A-M Systems), which were cut to a length of 15 cm. One end of each wire was soldered to a pin-type connector to interface with the device. The other end was exposed by removing 1 mm of the PFA coating. The exposed tip was then bent into a hook shape and placed inside the lumen of a 30-G hypodermic needle to facilitate intramuscular insertion. Two electrodes were implanted bilaterally in the EUS muscle, approximately 6–7 mm from the base of the bladder. Special care was taken to control the insertion depth to prevent urethral perforation or adjacent tissue damage. After insertion, the needle was removed, and gentle traction was applied to the electrode to verify its anchoring to the muscle tissue. Finally, a third reference electrode was percutaneously placed at the base of the subject’s tail via the same insertion procedure.

### Bladder catheterization

**Fig 3:**
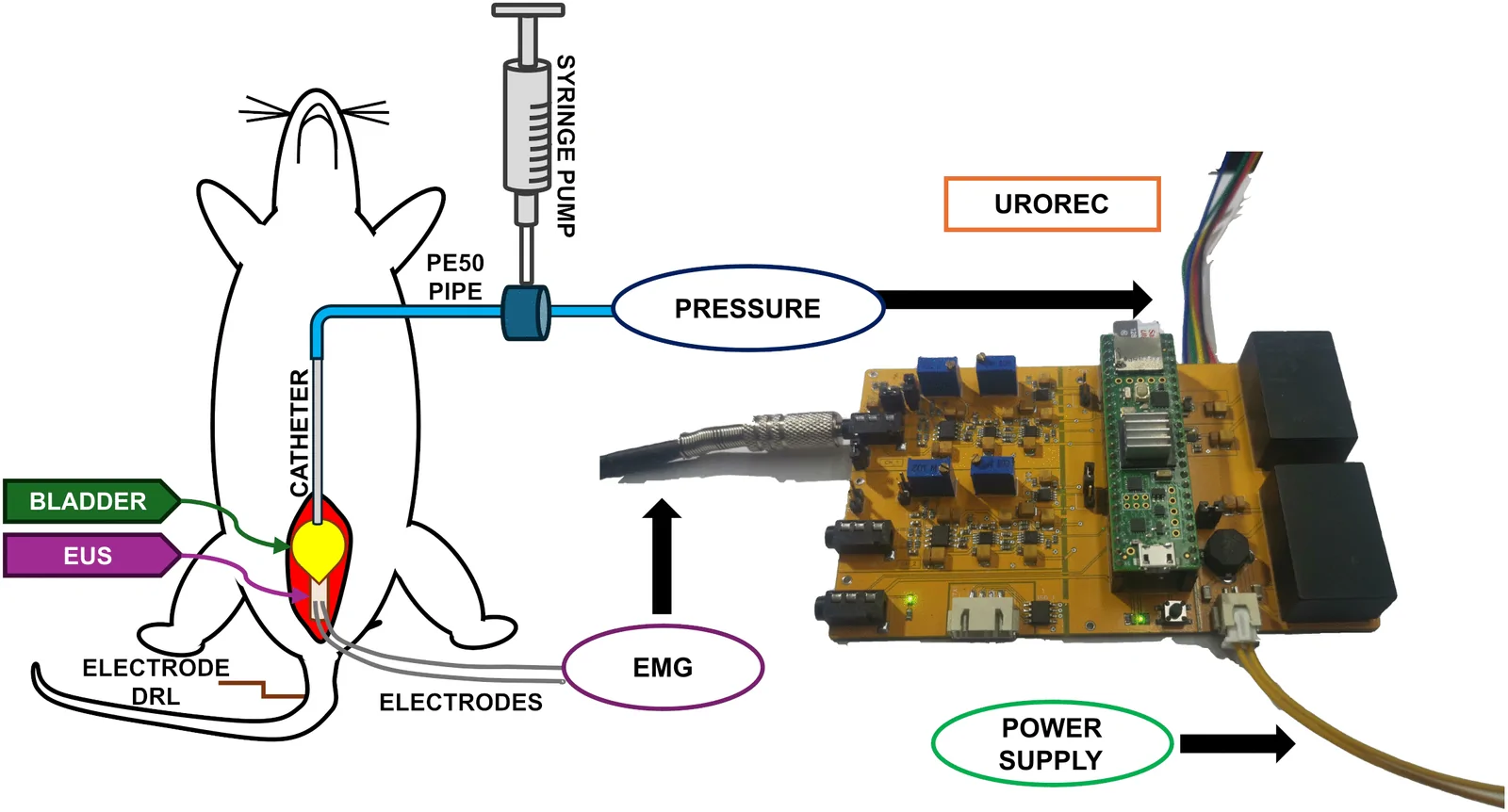
Schematic representation of the *in vivo* experimental setup. The system incorporates continuous intravesical infusion via a syringe pump, as well as pressure monitoring and EMG EUS recording via implanted electrodes.

The fluid system consisted of a 26-G catheter connected to a 15-centimeter polyethylene (PE-50) tube, a three-way stopcock, an infusion pump, and a pressure sensor. The experimental setup is shown in Fig 3. All the components were disinfected with 70% ethanol. The system was then purged with a saline solution to remove any air bubbles that could affect the accuracy of the pressure measurements [19].

With the bladder exposed, gentle traction was applied to the bladder dome tissue with dissecting forceps to facilitate the insertion of the catheter up to 10 mm deep. After the guide needle was removed, light pressure was applied to the bladder with a moistened cotton swab to force retrograde urine flow into the connector and ensure a continuous liquid column. The catheter was then connected to the PE-50 tube. Finally, the catheter was connected to the three-way stopcock, which served as a common interface for the infusion pump and digital pressure sensor. This catheterization technique was chosen instead of direct PE-50 tubing implantation because it minimizes detrusor muscle trauma and potential mechanical artifacts in the pressure signal [19].

### Urodynamic records

After the surgical procedures were complete, the anesthesia was switched from isoflurane to urethane (1.2 g/kg; Sigma-Aldrich) [10,22,26]. Half of the calculated dose was administered intraperitoneally (IP), and the inhaled anesthetic was reduced to 0.5%. Fifteen minutes later, the remaining dose was administered, and isoflurane was completely discontinued. A constant flow of oxygen was maintained for an additional 15–30 minutes to ensure oxygenation and respiratory stability during the transition. The depth of anesthesia was verified by assessing the absence of the paw withdrawal reflex to a nociceptive stimulus. If reflex activity was detected, supplemental doses of 10% of the initial dose were administered via IP. A 15-to-30-minutes observation period was subsequently conducted to reassess the anesthetic plane.

The electrodes and pressure sensor were subsequently connected to the system. The bladder was perfused at a constant flow rate of 0.3 mL/min until stable, and rhythmic voiding events were observed. This stabilization period lasted between 30 and 60 minutes, depending on the subject’s individual response. Once four to five consistent voiding cycles were achieved, data recording was initiated.

### Analysis parameters

Table 1 summarizes the urodynamic and EMG parameters most commonly used and reported in the literature for evaluating CMG and EUS-EMG characteristics in *in vivo* models [10,15,19,20,22,23,27–31].

**Table 1:** EUS-EMG and CMG parameters for healthy and pathological models in anesthetized rats.

Parameters	Description	Definition
**Cystometry**		
ACT	Actual Collection Time	The actual duration of the storage phase, calculated as ACT=ICI+2nd PD (s).
ICI	Inter-contraction Interval	The elapsed time between the end and the beginning of two consecutive micturition events (s).
VF	Voiding Frequency	The overall rate of voiding events is calculated over the total analysis duration (Contractions/min).
BCD	Bladder Contraction Duration	The total duration of detrusor contraction during the voiding phase BCD=1st PD+2nd PD (s).
1st PD	1st Phase Duration	The duration of the active voiding phase or primary contraction (s).
MVP	Maximum Voiding Pressure	The maximum peak of intravesical pressure during voiding (cmH_2_O).
BCP	Bladder Compliance	The distensibility of the bladder wall during the storage phase $BCP=\frac{infusio\_rate*(ICI/\text{6}\text{0})}{PT-RP}$.
PT	Pressure Threshold	The intravesical pressure that triggers the micturition reflex (cmH_2_O).
CPP	Closing Peak Pressure	The second pressure peak associated with the urethral sphincter closing reflex at the end of voiding (cmH_2_O).
2nd PD	2nd Phase Duration	The duration of the postmicturition tonic phase or secondary contraction (s).
RP	Resting Pressure	The lowest intravesical pressure immediately following a micturition event (cmH_2_O).
**Electromyography**		
Tonic Dur	Tonic Activity Duration	The duration of low-level tonic sphincter activity during the filling phase (s).
Inter-Burst Interval	Inter-Burst Interval	The elapsed time between the onset of two consecutive EUS phasic burst events (s).
EUS Burst Freq	Phasic Burst Frequency	The frequency of high-frequency EUS burst events during voiding (events/min).
Tonic RMS	Tonic RMS Amplitude	The root mean square (RMS) voltage amplitude of the sphincter during the filling or storage phase (mV).
Phasic Dur	Phasic Burst Duration	The duration of the explosive burst activity of the sphincter during the voiding phase (s).
Phasic AUC	Phasic Area Under the Curve	The area under the curve of the rectified EMG signal represents total muscle activation per event (mV * s).
Phasic RMS	Phasic RMS Amplitude	The RMS voltage amplitude of the sphincter during the burst/voiding phase (mV).

### Statistical analysis

The statistical analysis was performed using the R programming language (version 4.5.0) in the RStudio environment (version 2026.1.0) on a Windows 11 operating system. Before inferential analysis, the Shapiro–Wilk normality test was used to assess the distribution of the data. A paired Student’s t-test was performed on normally distributed data, which were reported as mean ± standard deviation (SD). Otherwise, the Wilcoxon signed-rank test was used, and the results were reported as the median and interquartile range (IQR). Statistical significance was initially evaluated at *p* < 0.05. To account for the increase in Type I error rates due to multiple comparisons across the cystometry and electromyographic parameters, the Benjamini-Hochberg False Discovery Rate correction procedure was applied. A formal a priori statistical power calculation was not performed due to the technical pilot validation and proof-of-concept nature of this hardware study. The sample size was determined based on institutional ethical minimization guidelines and established pilot benchmarks in the literature.

## Results

### Validation of the EMG electronic circuit

The preliminary benchtop characterization confirmed that the analog architecture provides the high fidelity and noise suppression required for EUS-EMG registration. A complete summary of the measured parameters and specific test conditions is presented in Table 2, and the experimental Bode plot, linearity regression curves, and mathematical filter scaling factors are detailed in S1 File. S1 Appendix, Note 2.

**Table 2:** Electrical characterization, in vivo signal integrity, and specific test conditions of the EMG circuit.

Parameters	Measured Value	Test Conditions
Mid-band Gain $A_{v}$	1070 V/V (60.6 dB)	Stable passband (40 Hz – 600 Hz).
Linearity ($R^{\text{2}}$)	0.9999	Regression analysis (Input range: $\text{2}-\text{6}\;mV_{pp}$).
Bandwidth (–3 dB)	5.1 Hz – 3.32 kHz	Sine sweep (0.5 Hz – 10 kHz), $V_{in}=\text{2}\;mV_{pp}$.
CMRR	98.4 dB	At 60 Hz sine wave ($V_{cm}=\text{3}.\text{1}\text{6}{\;V}_{pp}$), short-circuited inputs.
Input Dynamic Range	±3.0 mV	Linear output without clipping, ± 3.3 V supply.
Output Noise Floor	1.64 $mV_{rms}$	Active DRL, 10 kΩ skin impedance simulation.
Input-referred Noise (RTI)	1.53 $\mu V_{rms}$	Differential inputs were short-circuited, and the DRL was connected via a 10 kΩ resistor.
In vivo Raw SNR	12.33 ± 3.25 dB	Defined during the EUS-EMG burst phase versus the filling baseline floor.
In vivo Filtered SNR	19.26 ± 4.31 dB	Calculated post-processing signals. Achieved a 0% technical failure rate with zero unusable recordings or data corruption.

### Pressure sensor validation and calibration

The validation and calibration of the pressure module confirmed its suitability for continuous CMG acquisition. Within the selected physiological pressure range of 0–150 cmH_2_O, the sensor exhibited linear behavior, as evidenced by a coefficient of determination of $R^{\text{2}}$ = 0.9946. Similarly, evaluation of the load and unloading cycles revealed a maximum hysteresis of 0.29% at full scale. These results are consistent with the manufacturer’s specified theoretical accuracy band of ±0.25% for the combination of nonlinearity and hysteresis errors. These tests ensure that the system can accurately capture and record intravesical pressure dynamics, such as nonvoiding contractions and threshold pressures, in the murine model without introducing instrumental distortions.

### *In vivo* measurements

**Fig 4:**
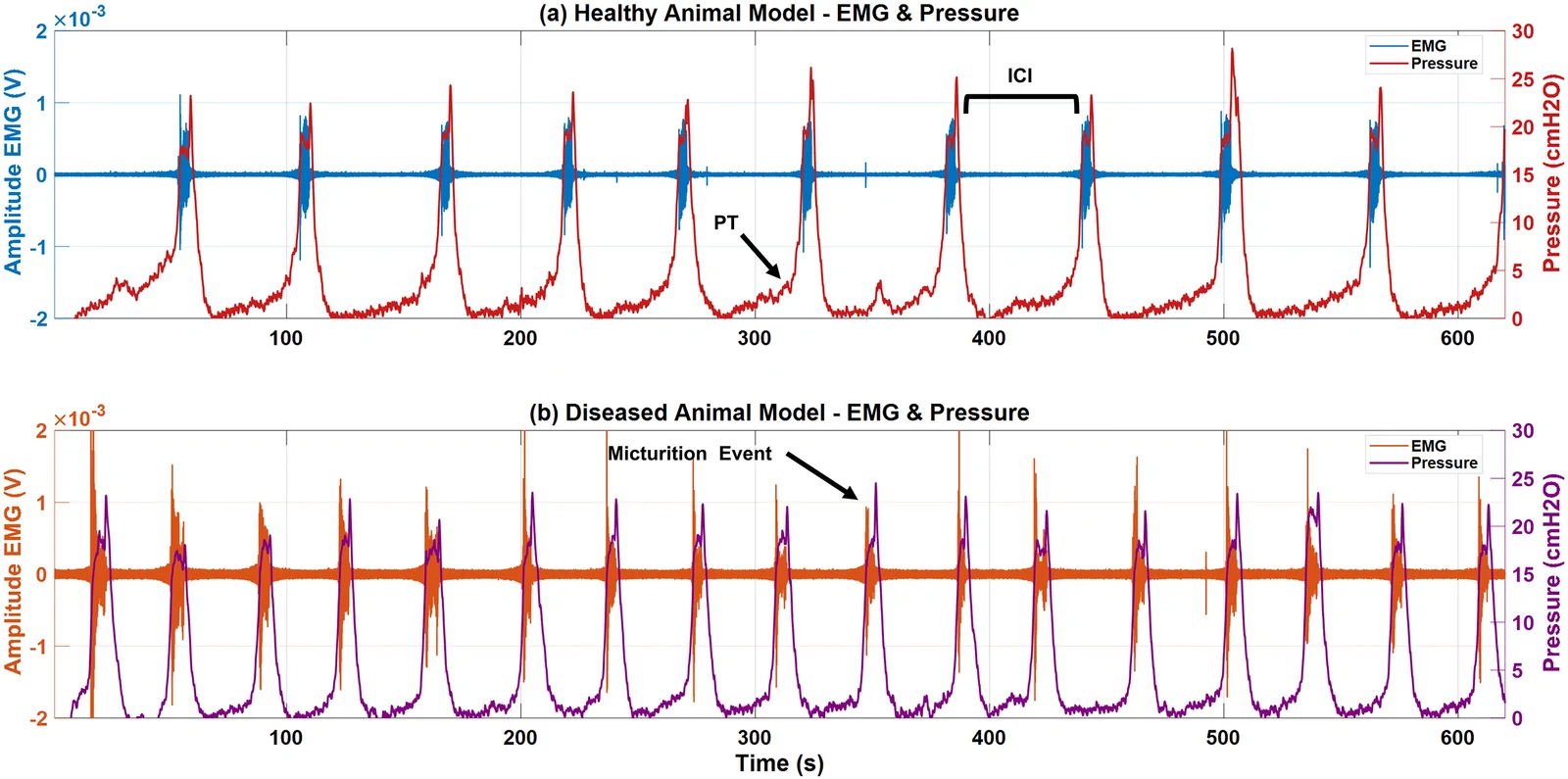
Simultaneous recording of EUS-EMG activity and intravesical pressure. (a) Representative EMG (blue) and pressure (red) traces from a healthy control model, displaying inter-contraction interval (ICI) and pressure threshold (PT). (b) Corresponding EMG (orange) and pressure (magenta) traces from a pathological model with an acetic acid-induced OAB, where the arrow indicates a micturition event.

All study subjects completed experimental procedures. Fig 4 shows representative EUS-EMG and intravesical pressure traces obtained from both healthy controls and pathological models. The EUS-EMG signal also remained stable at baseline, with no significant motion artifacts during the bladder filling phase. Across the entire in vivo dataset, UROREC demonstrated high signal integrity with a mean raw SNR of 12.33 ± 3.25 dB, which significantly improved to 19.26 ± 4.31 dB post-filtering (Table 2). The platform achieved a 100% technical success rate, with zero recording sessions or data blocks excluded due to hardware saturation or signal corruption.

**Fig 5:**
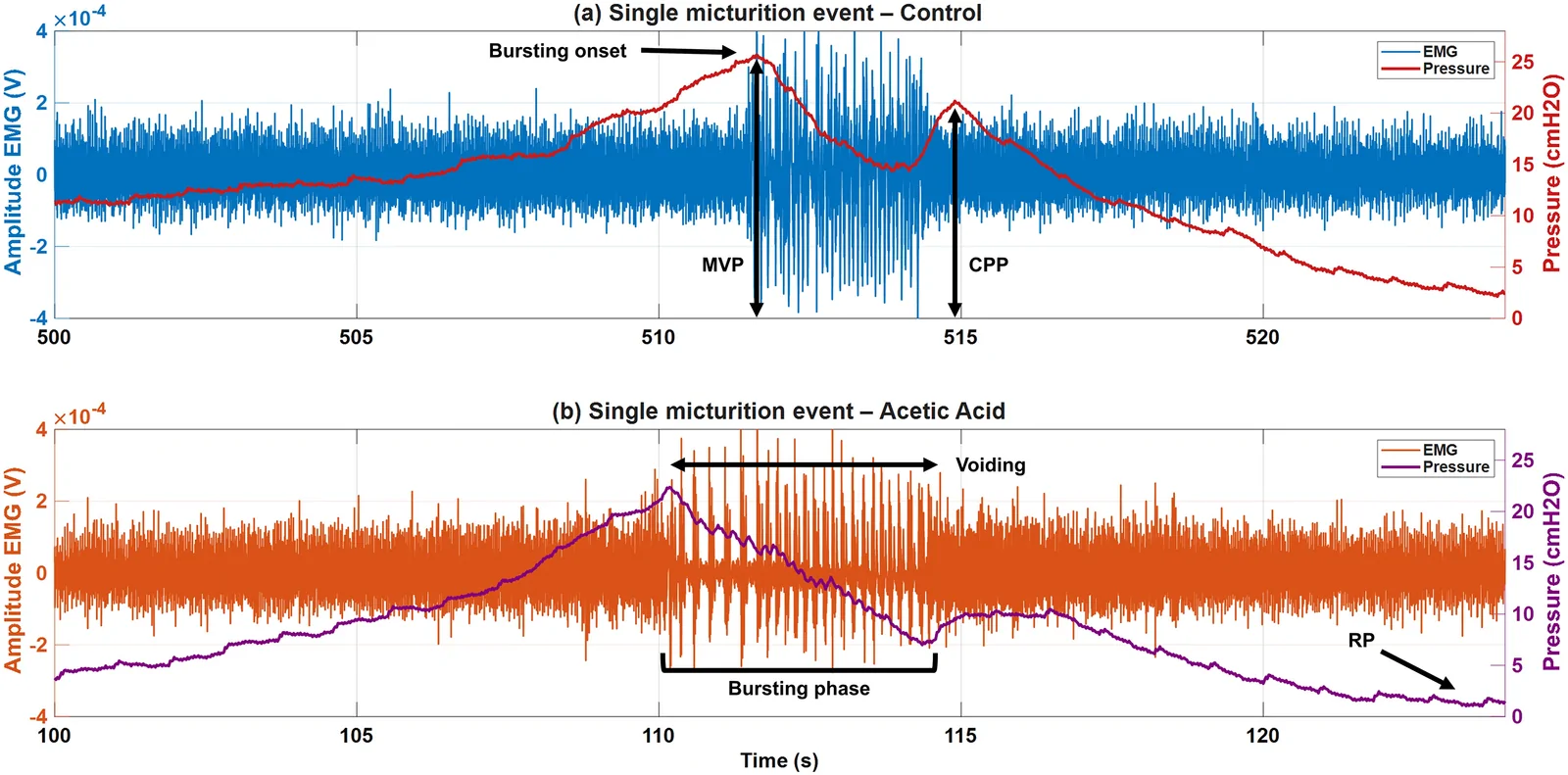
Details of micturition events. (a) Synchronous EUS-EMG (blue) and pressure (red) in a control model, highlighting the bursting onset, maximum voiding pressure (MVP), and closing peak pressure (CPP). (b) Equivalent traces (orange and purple) in the acetic acid model. High-resolution views reveal bursting phase activity and rapid pressure oscillations resulting from chemical irritation, followed by a return to the resting pressure (RP).

Fig 5 provides a detailed view of a single micturition event, clearly displaying the characteristic patterns of phasic EUS muscle activity bursts. The intravesical pressure trace shows a steady increase before the EMG bursts, indicating the onset of bladder contractions. A decrease in pressure was subsequently observed during the voiding phase. High-frequency oscillations in the recording allowed for the precise identification of individual contractions within the burst activity.

### Comparative analysis: Healthy vs. pathological models

A paired quantitative analysis was performed to evaluate functional changes before and after the pathological model was implemented. The intravesical pressure and EMG records from the EUS were processed to extract the previously defined urodynamic and neuromuscular parameters, which were presented in Table 1. A summary of the results obtained from the statistical tests is presented in Table 3.

**Table 3:** Comparison of healthy vs. pathological models (N = 15). The data are presented as the mean ± SD or median and IQR.

Parameters	Healthy (Mean ± SD or Median [IQR])	Pathological (Mean ± SD or Median [IQR])	*p*
**Cystometry**			
ACT (s)	72.8180 ± 27.0912	41.6258 ± 17.3714	**< 0.001** ^*^
ICI (s)	66.1230 ± 28.2483	34.2131 ± 16.5616	**< 0.001** ^*^
VF (events/min)	0.7500 ± 0.2999	1.3073 ± 0.4924	**0.0014** ^*^
BCD (s)	22.0000 [19.1188 - 32.1782]	16.8272 [15.1291 - 19.4769]	**0.0041** ^*^
1st PD (s)	21.8338 ± 15.3117	10.6431 ± 5.0671	**0.0047** ^*^
MVP (cmH_2_O)	32.2946 [29.2174 - 46.4011]	27.6741 [24.4530 - 35.7095]	**0.0049** ^*^
BCP (mL/ cmH_2_O)	0.0550 ± 0.0248	0.0435 ± 0.0213	**0.0141** ^*^
PT (cmH_2_O)	7.4254 ± 2.8734	5.6890 ± 3.3735	**0.0270** ^*^
CPP (cmH_2_O)	33.7967 ± 11.9977	29.2412 ± 11.5691	**0.0324** ^*^
2nd PD (s)	6.7658 ± 3.8212	7.2535 ± 2.9060	0.6780
RP (cmH_2_O)	0.8901 [0.7487 - 1.0410]	0.8867 [0.7549 - 1.1544]	0.9321
**Electromyography**			
Tonic Dur (s)	84.0666 ± 37.7604	43.7859 ± 18.6735	**< 0.001** ^*^
Inter-Burst Interval (s)	93.1495 ± 37.3901	51.3246 ± 18.8786	**< 0.001** ^*^
EUS Burst Freq (events/min)	0.7520 ± 0.3071	1.2807 ± 0.4074	**< 0.001** ^*^
Tonic RMS (mV)	0.0206 ± 0.0134	0.0241 ± 0.0152	0.0581
Phasic Dur (s)	7.4617 [6.1553 - 9.6812]	6.4832 [5.6659 - 9.4767]	0.2220
Phasic AUC (mV·s)	0.6056 ± 0.4548	0.5603 ± 0.6945	0.7282
Phasic RMS (mV)	0.1175 ± 0.1035	0.1199 ± 0.1325	0.8909

Data were evaluated using a paired Student’s t-test or a Wilcoxon signed-rank test. Significant differences (*p* < 0.05) are indicated by an asterisk (*) and bold values. Adjustments for multiple comparisons were performed using the Benjamini-Hochberg False Discovery Rate procedure across all 18 evaluated parameters.

## Discussion

Recording devices in LUT research are essential for understanding neural control mechanisms and evaluating new treatments [32,33]. However, significant gaps remain in our understanding of the complex physiological interactions that regulate micturition [34], highlighting the need for accurate, accessible instrumentation. This study introduces and validates UROREC, a system designed to monitor bladder pressure and EUS-EMG in preclinical models simultaneously. By using commercial components, UROREC adheres to methodological standards for sampling frequencies and resolutions, enabling high-fidelity urodynamic analysis.

UROREC’s technical characterization demonstrated robustness comparable to commercial instrumentation. The 98.4 dB CMRR and low RTI noise ensure high-fidelity EMG recordings in anesthetized rats, where microvolt-range EUS signals are highly susceptible to 60 Hz interference. The validated 5.1 Hz–3.32 kHz bandwidth captures all relevant spectral content of EUS motor unit discharges. Furthermore, a linearity of $R^{\text{2}}$ = 0.9999 within ±3.0 mV preserves the morphology of tonic and phasic contractions, which is crucial for quantifying subtle EMG variations across pathological states or anesthetic regimens. Continuous CMG accuracy is supported by the pressure module’s low hysteresis, which ensures a reliable return to baseline and prevents compliance errors in the infusion lines. Unlike many biomedical prototypes, UROREC was rigorously validated *in vivo* using standard protocols [19,26], successfully quantifying 18 EUS-EMG and CMG parameters consistent with previous studies [10,15,19,20,22,23,27–31]. Crucially, to address the critical distinction between true measurement accuracy and internal consistency, the absolute baseline values quantified by UROREC in healthy controls were directly benchmarked against metrics established by high-end commercial workstations. For instance, the baseline Maximum Voiding Pressure (MVP) of 32.2946 [29.2174–46.4011] cmH_2_O and Pressure Threshold (PT) of 7.4254 ± 2.8734 cmH_2_O recorded by UROREC fall strictly within the classic physiological ranges reported for urethane-anesthetized female Wistar rats, which typically span 20–35 cmH_2_O for MVP and 5–10 cmH_2_O for PT in gold standard urodynamic studies [19,20,30]. This numerical correlation with established benchmarks demonstrates that UROREC does not merely track isolated internal trends or relative changes but successfully translates its metrological benchtop calibration into accurate physical units within the live environment. A key advantage of UROREC is its independence from specialized workstations and bulky power supplies. Its architecture supports real-time monitoring, open-source signal processing, and direct integration into established experimental procedures without altering gold-standard urodynamic protocols [10,33].

In the context of the operational value of UROREC within current preclinical research, its architecture was systematically benchmarked against current market-leading commercial equipment in a workstation format and general-purpose open-source biosignals acquisition frameworks [10,33], as shown in Table 4. Although high-end commercial platforms offer high reliability, their proprietary software and closed data formats restrict custom signal analysis and real-time integration with specialized research workflows. Conversely, while general open-source boards [35] provide financial accessibility, they lack specialized EUS-EMG and cystometry requirements, meaning they cannot safely handle synchronous intravesical pressure and electromyographic signals simultaneously. UROREC bridges this technological gap by integrating a purpose-built preclinical conditioning stage into a single compact board footprint, reducing the economic barrier while maintaining the necessary specifications to perform replicable experimentation and research. Comprehensive details regarding the component specifications, unit costs, and hardware accessibility guidelines are provided in S4 Note.

**Table 4:** Technical and economic benchmark of UROREC against commercial and open-source systems.

Feature	Commercial Workstation (BIOPAC Systems, AD Instruments Power La) [10,33]	Open-Source Platform (OpenBCI Cyton) [35]	UROREC (This Work)
Accessibility	Low: Closed-source software, proprietary formats, expensive annual licenses.	High: Fully open-source software and hardware documentation.	High: Fully open-source schematics, hardware, and software.
Preclinical Specialization	High/Rigid: Specialized but requires modular expansion cards for dual CMG + EMG.	Low: Designed for Neural interfaces (EEG); lacks integrated pressure sensor stages.	High/specialized: Purpose-built specifically for synchronous urodynamics (CMG + EUS-EMG).
Sampling Rate and Resolution	200 ksps/ 16 bit.	250 sps to 16 ksps/ 24 bit.	20 ksps/ 12 bit.
Isolation	Optical/ Medical Grade Galvanic ISO.	None (Requires external battery operation for basic safety).	Galvanic isolation via DC-DC converters, battery, and digital isolators.
Compactness	Low: Heavy, rigid cart or desktop workstations; restricted to fixed laboratory benches.	Medium: Compact board but requires external clustering for pressure transducers.	High: Compact, single-board architecture with local micro-SD storage.
Cost	High: Can exceed $10,000 USD per full configuration plus specialized software licensing.	Moderate: Around $1000 USD after adding necessary analog modules.	Low: Around $273 USD with all necessary components to perform CMG and EUS-EMG.

The results of this study validated the sensitivity of UROREC in detecting and quantifying pathophysiological changes in LUT research. In the chemically irritated bladder model, instilling acetic acid resulted in a statistically significant reduction in both ICI and ACT (*p* < 0.001). These results are consistent with the occurrence of bladder hyperactivity induced by chemical irritation, a phenomenon widely reported in the literature [10,14,27]. Additionally, significant alterations in BCP (*p* = 0.014) and MVP (*p* = 0.0049) were detected. The reduction in MVP observed in our pathological group is consistent with Yoshiyama’s findings in murine models, in which acid irritation markedly decreased bladder pressure during the micturition reflex [27]. These results suggest potential reflex inhibition of bladder contractility or alterations in urethral outlet resistance. These phenomena vary in rodents and depend on experimental conditions or anesthesia [10,19]. Using recordings from UROREC, we quantified a significant decrease in PT (*p* = 0.027), which was associated with a reduction in the micturition reflex trigger threshold [10,12]. Additionally, the analysis revealed a significant decrease in the 1st PD in the pathological group (*p* = 0.0047), likely due to the experimental model used. As reported in [27], decerebration animal models enable the isolation of the micturition reflex without depression. Our urethane anesthesia model preserved micturition reflexes but introduced pharmacological modulation of the efficiency and activation thresholds of recorded events [19,26].

In contrast to previous studies and conventional methodologies that indirectly quantify sphincter activity through high-frequency oscillations in intravesical pressure or visual observation of urethral dribbling [10,16,19], the UROREC system directly quantifies the EMG activity of the EUS synchronized with the CMG. This is evident in the reduction in the CMG ICI accompanied by the EMG IBI (*p* < 0.001) and Tonic Dur. This confirms the device’s ability to monitor bladder-sphincter coordination. This feature offers a technological advantage for investigating the neurophysiology of micturition reflexes in murine models. On the other hand, the device enabled us to identify parameters that remained stable compared with those of the pathological model. Specifically, the Phasic RMS, Phasic AUC, and Phasic Dur did not show statistically significant variations (*p* > 0.05). These findings confirm that acute intravesical acid irritation is strictly localized to the bladder urothelium, modulating the frequency of the micturition reflex through afferent sensitization without inducing direct muscular or neuromuscular alterations in the integrity of the EUS [10,27]. Figs 4 and 5, together with the collected data, enable us to perform visual and quantitative analyses of these behaviors.

The present study confirms the sensitivity of UROREC in characterizing the proposed pathological model in female rats. However, we acknowledge that excluding male specimens limits our ability to evaluate sex-specific urodynamic behaviors. For example, phenomena such as urethral irritability and postvoid dribbling, which are characteristic of bladder irritability in male rodents, were beyond the scope of this study. Additionally, variations in voiding event duration compared with those in awake murine models suggest that the anesthetic regimen modulates reflex arc efficiency. Consequently, future studies using the UROREC platform will focus on comparative analyses of sexual dimorphism and the impact of different anesthetic protocols. The inclusion of male models in future research will further validate the device’s ability to detect subtle, sex-specific functional alterations in LUT physiology. Furthermore, it is important to note that while the acute chemical irritation model provided a highly predictable and reliable baseline for initial hardware validation, it represents an acute irritative state rather than a true chronic or neurogenic bladder pathophysiology. Therefore, this pilot validation must be acknowledged as a foundational and mandatory step required to establish the platform’s operational sensitivity before planning and deploying future chronic urological trials. Additionally, a limitation of this initial pilot phase is that simultaneous, side-by-side parallel recordings with a commercial gold-standard workstation were not performed. Although UROREC’s absolute quantified parameters were validated by benchmarking them against established historical metrics from the literature, direct real-time cross-validation with proprietary equipment remains a key objective for future experimental trials. While a cohort of 15 subjects represents a baseline size common in preliminary hardware characterizations, this study was explicitly designed as a paired pilot validation evaluation. One of the primary methodological contributions of this pilot phase is to generate the preliminary statistical datasets reported herein, which are strictly necessary to conduct accurate a priori statistical power calculations in future large-scale chronic or pharmacological trials utilizing the UROREC platform.

To facilitate replication, UROREC supports a dual-assembly framework tailored to different laboratory capabilities (S1 File. S1 Appendix, Note 4). Non-engineering groups can utilize modern commercial assembly services, completely eliminating the need for manual soldering. Conversely, for laboratories seeking to optimize budgets or leverage institutional resources, the system can be hand-assembled using basic electronic prototyping tools. This open-hardware approach not only significantly lowers financial entry barriers but also promotes valuable interdisciplinary collaboration during the deployment phase. In this context, UROREC is established as a robust, accessible, and cost-effective alternative to commercial workstations. Its implementation provides laboratories with a tool for rigorous urodynamic analysis, enabling groups with limited resources to conduct research that aligns with current reproducibility standards, such as the ARRIVE 2.0 guidelines, while reducing operating costs. UROREC was specifically designed to advance research in the LUT.

## Conclusion

This study presented and validated the UROREC system for the simultaneous monitoring of EUS EMG activity and CMG in murine models, such as Wistar rats. The device has demonstrated high sensitivity in distinguishing between signals generated by healthy and pathological states. It adheres strictly to gold standard protocols without requiring modifications to established surgical procedures. UROREC fills a critical gap in specialized instrumentation for preclinical research by combining robust data acquisition with portability and affordability. Future applications will expand the use of this system to further study the pathophysiology of UI and evaluate the efficacy of pharmacological and alternative therapeutic interventions. This will establish UROREC as a valuable, accessible tool for advancing LUT research, providing a scalable system architecture that can be adapted for other medical applications requiring synchronous biopotential and pressure monitoring.

## Supporting information

S1 File S1 Appendix. (DOCX)
