# Supplementary material for: UROREC: A cystometry and electromyography recording system for analyzing urinary incontinence parameters in rat models
Source: PLoS One. 2026 Aug 26;21(8):e0356999. doi: 10.1371/journal.pone.0356999 (PMC13513990; doi:10.1371/journal.pone.0356999)
Supplement: Supplementary file 1 [file pone.0356999.s001.docx]

S1 Appendix

Note 1

Electromyography acquisition circuit

The circuit includes components 0603 package resistors with 0.1% and capacitors with 10% tolerance, an INA828ID instrumentation amplifier, and OPA2145IDGKT precision operational amplifiers (Texas Instruments). S1 Fig. shows a schematic of the implemented electronic circuit.

**S1 Fig.** **Schematic diagram of the analog signal conditioning stage.** (a) High-pass filter and AC-coupled input protection [1], (b) instrumentation amplifier [2], (c) active Sallen-Key low-pass filter, $f_{c}\approx3 kHz$ [3–5], (d) gain stage with noninverting amplifier, (e) DC offset reference generation, (f) final output level shifter, (g) driven right leg (DRL) active noise suppression circuit [6].

The electromyography (EMG) circuit was physically characterized in a laboratory setting to evaluate its electrical performance metrics against physical voltage standards. Parameters such as the midband gain, linearity, frequency bandwidth, common-mode rejection ratio (CMRR), input dynamic range, output noise floor, and input-referred noise (RTI) were measured [7–9]. The characterization process was divided into four stages:

1. *Frequency response and bandwidth:* These parameters were determined by performing a frequency sweep from 0.5 Hz to 10 kHz and applying a sine wave signal with a constant amplitude of $2.0 mV_{pp}$ to the differential input. The output voltage was recorded throughout the sweep, and the -3 dB cutoff frequency was calculated.
2. *Linearity and dynamic range:* These parameters were evaluated by applying a 100 Hz, $2.0 mV_{pp}$ sine wave signal to the differential input. The amplitude was increased in $1.0 mV_{pp}$ steps and recorded until output saturation occurred. The effective differential gain and the coefficient of determination ($R^{2}$) were calculated via linear regression analysis of the input versus output data. The dynamic input range was defined as the maximum amplitude of the input signal before the circuit output was saturated or distorted.
3. *Common Mode Rejection Ratio (CMRR):* The circuit's ability to suppress power line interference was evaluated. The differential inputs were short-circuited and connected to a 60 Hz, $3.0 mV_{pp}$ sine wave signal.
4. *Input-referred noise (RTI):* Electronic noise generated by system components was evaluated by short-circuiting the differential inputs. The DRL was connected through a 10 kΩ resistor to simulate the electrode-skin interface impedance. The output signal was recorded, and the RTI noise of the system was calculated.

Note 2

Validation of the EMG Electronic Circuit

The validation process focused on characterizing the EMG circuit's electrical performance. The interference suppression evaluation yielded a CMRR of 98.4 dB at 60 Hz. The circuit exhibited a stable frequency response with a midband gain of 1070 V/V, as shown in S2 Fig. The filter's natural frequency, $f_{0}$, was set to 4.8 kHz and adjusted throughout the characteristic scaling factor of the Sallen-Key topology, Q = 0.5. This yielded effective cutoff frequencies of -3 dB at 5.1 Hz and 3.32 kHz. Linearity was confirmed by an $R^{2}$ coefficient of 0.9999, which ensured signal fidelity within a dynamic input range of ±3.0 mV. Finally, the noise analysis yielded an RTI of $1.53 \mu V_{RMS}$.

**S2 Fig.** **Electrical characterization of the EMG circuit.** (a) Bode diagram illustrating the passband and the lower and upper -3 dB cutoff frequencies. (b) Linearity and dynamic range analysis, which delimits the linear operating region from the amplifier saturation zone. The regression of 992 V/V versus the maximum midband gain of 1070 V/V shows the expected theoretical attenuation due to curvature of the Sallen-Key filter.

Note 3

Cystometry Acquisition Circuit

The sensor output pressure was determined via Equation (1), which was provided by the manufacturer.

$$P_{out}=\frac{\left( O_{1}-O_{min} \right)*\left( P_{max}-P_{min} \right)}{O_{max}-O_{min}}+P_{min}$$

(1)

where $P_{out}$ is the calculated output pressure and $O_{1}$ is the digital pressure output value. $P_{max}$ and $P_{min}$ represent the maximum and minimum operating pressure ranges of the sensor, respectively. $O_{max}$ and $O_{min}$ are the digital output values corresponding to the maximum and minimum pressures, respectively. The sensor's original output units are pounds per square inch (PSI), which are subsequently converted to centimeters of water (cmH₂O) via the conversion factor described in Equation (2).

$$P_{cmH_{2}O}= P_{out}*70.307$$

(2)

To facilitate connection and disconnection, the sensor was mounted on a custom-designed FR-4 printed circuit board (PCB). The assembly incorporates a protective housing that was 3D-printed using a polylactic acid (PLA) filament. It also includes a 15 cm wiring interface. Before *in vivo* experimentation, tests were performed to verify the linearity of the sensor. This was done via a calibration based on the measurement of a controlled hydrostatic water column, establishing an absolute physical calibration standard. These tests followed established bench characterization protocols for bladder pressure sensors. These protocols are similar to those reported in [10–12]. Controlled hydrostatic pressures ranging from 0 to 150 cmH₂O in 5 cmH₂O increments were applied, and the sensor's hysteresis and linearity were evaluated. The linearity coefficient was then calculated to confirm the sensor's ability to capture pressure change dynamics during micturition events in the selected murine model.

Note 4

Digital Processing and Interface Unit

The central processing unit is a Teensy 4.1 board; it has a 600 MHz ARM Cortex-M7 microcontroller (NXP iMXRT1062), a 12-bit analog-to-digital converter (ADC), a microSD card slot, 8 MB of flash memory for program storage, and 1 MB of RAM. These features enable efficient data storage and high-speed writing to the SD card. The proposed device uses the Direct Memory Access (DMA) controller to manage the simultaneous acquisition of both EUS-EMG and cystometry (CMG) data. The DMA controller facilitates direct data transfer to memory without CPU intervention.

To maintain graphical fluidity, the EMG signal was downsampled to 4 ksps, whereas the pressure signal was downsampled to 45 sps. This strategy significantly reduces the computational load required for data transmission and unit conversion. Importantly, this optimization prevented interference with the high-speed raw data logging managed by the DMA controller in the background, ensuring continuous and smooth graphical output. The system was designed to operate with either an external 9 V power supply based on lithium-ion batteries or a connection to the main power supply via a power adapter. (MPOWER model MDD06F-05). Next, low-noise linear regulators derived ±3.3 V rails dedicated to the analog front–end operational amplifiers. The combination of this symmetrical power supply and a 1.65 V DC offset applied during the final conditioning stage allowed the output signals to remain strictly within the 0 V to 3.3 V dynamic range. This configuration ensured direct voltage-level compatibility between the analog stage and the ADC input, prevented signal saturation, and maximized the resolution of the digital conversion. The assembled device is shown in S3 Fig.

**S3 Fig.** Top view of the custom-made main board. This design integrates the analog front end, microcontroller unit, and galvanically isolated power supply modules onto a single PCB. It is designed for portability and reduced noise interference.

Note 4

Bill of Materials and Hardware Accessibility

As itemized in S1 Table, the definitive baseline cost for replicating a single UROREC unit via the in-house hand-assembly pathway is $272.236 USD (excluding local shipping, customs, and taxes). This comprehensive cost matrix includes the commercial fabrication of the custom 2-layer FR-4 printed circuit board via prototype manufacturing services (such as JLCPCB), as well as all specialized components. By leveraging manual component population and institutional electronic prototyping tools, laboratories can completely bypass the premium overhead associated with commercial printed circuit board assembly (PCBA). Even when factoring in loose component retail pricing, this total architecture cost represents a percentage of the financial investment required for high-end, proprietary commercial urodynamic workstations. This data robustly substantiates UROREC’s claim as an accessible, economically scalable, and highly reproducible alternative for preclinical instrumentation of the lower urinary tract.

**S1 Table.** Bill of materials and costs.

| Part Number  (Mouser Electronics) | Description | Designator | Quantity | Unit Price |  |
| --- | --- | --- | --- | --- | --- |
| ADP7183ACPZN3.3-R7 | Integrated Circuit | -3.3V | 1 | $6.9 | $6.90 |
| T491A104K050AT7280 | Capacitor Polarized | 0.1uF_TC1, 0.1uF_TC2, 0.1uF_TC5, 0.1uF_TC6, 0.1uF_TC7, 0.1uF_TC8, 0.1uF_TC9 | 7 | $0.702 | $4.914 |
| 3296W-1-102LF | Variable Resistor | 1KR_T1, 1KR_T2, | 2 | $3.13 | $6.26 |
| 3296W-1-103LF | Variable Resistor | 10K_T1, 10K_T2 | 2 | $3.11 | $6.22 |
| TLV75533PDYDR | Integrated Circuit | 3.3V | 1 | $0.39 | $0.39 |
| GCM31CD71H106KE36K | Capacitor | 10uF_C1, 10uF_C2 | 2 | $1.05 | $2.10 |
| T495B106M020ATE1K0 | Capacitor Polarized | 10uF_TC1, 10uF_TC2, 10uF_TC3, 10uF_TC4, 10uF_TC5, 10uF_TC6, 10uF_TC7, 10uF_TC8, 10uF_TC9, 10uF_TC11, 10uF_TC12, 10uF_TC13, 10uF_TC14, 10uF_TC15, 10uF_TC16, 10uF_TC17, 10uF_TC18, 10uF_TC19, 10uF_TC20, 10uF_TC21, 10uF_TC10 | 21 | $1.5 | $31.50 |
| 502352-0201 | Connector | 12V | 1 | $1.13 | $1.13 |
| CMS2-9-R | Filters | CMC_1 | 1 | $3.19 | $3.19 |
| 90120-0122 | Headers | CON1, DRL, DRL_1, DRL_2, DRL_GND1, DRL_GND2, POT1, POT2, EMG1, EMG2 | 10 | $0.335 | $3.350 |
| MFU0805FF00500P500 | Fuse | FUS1 | 1 | $0.624 | $0.624 |
| INA828IDR | Integrated Circuit | INST_1, INST_2 | 2 | $8.18 | $16.36 |
| SM0603SRC | LED | LEDR_1 | 1 | $0.845 | $0.845 |
| SM0603GCL | LED | LEDG_1, LEDG_2 | 2 | $0.754 | $1.508 |
| SJ2-3573A-SMT-TR | Connector | J1, J1P, J2 | 3 | $1.54 | $4.62 |
| ADUM1201WURZ-RL7 | Integrated Circuit | ISO_1 | 1 | $14.90 | $14.90 |
| 502352-0601 | Connector | PRED_DIG | 1 | $1.51 | $1.51 |
| OPA2145IDGKR | Integrated Circuit | PRES_1, PRES_2, PRES_3, PRES_4, PRES_5, PRES_6, PRES_7 | 7 | $6.10 | $42.70 |
| MDD06F-05 | Power Supply | PS+/-5V | 1 | $23.25 | $23.25 |
| MDS06F-05 | Power Supply | PS+5V | 1 | $21.36 | $21.36 |
| 502352-0401 | Connector | RX1/TX1 | 1 | $1.29 | $1.29 |
| PTS647SK50SMTR2LFS | Switch | S1 | 1 | $0.39 | $0.39 |
| DEV-16771 | RT1062 Teensy 4.1 series ARM® Cortex®-M7 MPU Embedded Evaluation Board | TEE | 1 | $31.50 | $31.50 |
| CRCW06031M00DHEAP | Resistor | 1M_R1, 1M_R2, 1M_R3 | 3 | $0.286 | $0.858 |
| RT0603BRB071K8L | Resistor | 1.8K_R1,  1.8K_R2 | 2 | $0.403 | $0.806 |
| RT0603BRE072KL | Resistor | 2K_R1, 2K_R2 | 2 | $0.156 | $0.312 |
| RGC1/16K3304BTP | Resistor | 3.3M_R1, 3.3M_R2, 3.3M_R3, 3.3M_R4, 3.3M_R5, 3.3M_R6, 3.3M_R7, 3.3M_R8 | 8 | $0.286 | $2.288 |
| RT0603BRD075K1L | Resistor | 5.1K_R3, 5.1K_R4 | 2 | $0.13 | $0.26 |
| RT0603BRD077K5L | Resistor | 7.5K_R1, 7.5K_R2 | 2 | $0.13 | $0.26 |
| RT0603BRE0710KL | Resistor | 10K_R1, 10K_R3, 10K_R4, 10K_R5, 10K_R6, 10K_R8, 10K_R9, 10K_R10, 10K_R11, 10K_R12, 10K_R13, 10K_R14, 10K_R15, 10K_R16, 10K_R17, 10K_R18, 10K_R19, 10K_R20 | 18 | $0.13 | $2.34 |
| RT0603BRE0718KL | Resistor | 18K_R1, 18K_R2 | 2 | $0.13 | $0.26 |
| RT0603BRD0747RL | Resistor | 47_R1, 47_R2 | 2 | $0.13 | $0.26 |
| RT0603BRE07100KL | Resistor | 100K_R1 | 1 | $0.13 | $0.13 |
| RT0603BRD07330RL | Resistor | 330_R1, 330_R2, 330_R3, 330_R4 | 4 | $0.13 | $0.52 |
| RT0603BRD131KL | Resistor | RG_1, RG_2, RR_1 | 3 | $0.13 | $0.39 |
| CL10B104KO8NFNC | Capacitor | 0.1uF_C1, 0.1uF_C2, 0.1uF_C3, 0.1uF_C4, 0.1uF_C5, 0.1uF_C6, 0.1uF_C7, 0.1uF_C8, 0.1uF_C9, 0.1uF_C10, 0.1uF_C11, 0.1uF_C12, 0.1uF_C13, 0.1uF_C14  100nF_C1, 100nF_C2, 100nF_C3, 100nF_C4 | 18 | $0.13 | $2.34 |
| GCD188R71H102KA01D | Capacitor | 1nF_C1 | 1 | $0.13 | $0.13 |
| C1608X7R1C105K080AC | Capacitor | 1uF_C1, 1uF_C2, 1uF_C4, 1uF_C5, 1uF_C6, 1uF_C7 | 6 | $0.156 | $0.936 |
| CL10A475KQ8NNNC | Capacitor | 4.7uF_C1, 4.7uF_C2 | 2 | $0.13 | $0.26 |
| GCJ188R71C103KA01D | Capacitor | 10nF_C1, 10nF_C2, 10nF_C3, 10nF_C4, 10nF_C7 | 5 | $0.325 | $1.625 |
| ABP2LANT015PGSA3XX | Pressure Sensor | PR_SEN | 1 | $21.95 | $21.95 |
| PCB_1 | Manufacturing in JLCPCB, 5 pieces minimum (cost before shipping and taxes) | PRESSURE SENSOR | 1 | $2.10 | $2.10 |
| PCB_2 | Manufacturing in JLCPCB, 5 pieces minimum (cost before shipping and taxes) | UROREC | 1 | $7.40 | $7.40 |
| **Total Cost (USD)** |  |  |  |  | **$272.236** |

Prices are based on single-unit retail costs from major distributors as of 2026.

References

1.	Spinelli EM, Pallas-Areny R, Mayosky MA. AC-coupled front-end for biopotential measurements. IEEE Transactions on Biomedical Engineering. 2003;50: 391–395. doi:10.1109/TBME.2003.808826

2.	Smit HW, Verton K, Grimbergen CA. A Low-Cost Multichannel Preamplifier for Physiological Signals. IEEE Transactions on Biomedical Engineering. 1987;BME-34: 307–310. doi:10.1109/TBME.1987.326092

3.	Sallen RP, Key EL. A practical method of designing RC active filters. IRE Transactions on Circuit Theory. 1955;2: 74–85. doi:10.1109/TCT.1955.6500159

4.	Moazzam Z, Yoo PB. Frequency-dependent inhibition of bladder function by saphenous nerve stimulation in anesthetized rats. Neurourology and Urodynamics. 2018;37: 592–599. doi:10.1002/nau.23323

5.	Chang H-Y, Cheng C-L, Chen J-JJ, de Groat WC. Serotonergic drugs and spinal cord transections indicate that different spinal circuits are involved in external urethral sphincter activity in rats. American Journal of Physiology-Renal Physiology. 2007;292: F1044–F1053. doi:10.1152/ajprenal.00175.2006

6.	Winter BB, Webster JG. Driven-right-leg circuit design. IEEE Transactions on Biomedical Engineering. 1983;BME-30: 62–66. doi:10.1109/TBME.1983.325168

7.	Webster JG. Medical instrumentation: application and design. John Wiley & Sons; 2009.

8.	Merletti R, Hermens HJ. Detection and Conditioning of the Surface EMG Signal. Electromyography. 2004. pp. 107–131. doi:10.1002/0471678384.ch5

9.	Koutsoftidis S, Barsakcioglu DY, Petkos K, Farina D, Drakakis EM. Myolink: A 128-Channel, \text18 nV/\sqrt\textHz, Embedded Recording System, Optimized for High-Density Surface Electromyogram Acquisition. IEEE Transactions on Biomedical Engineering. 2022;69: 3389–3396. doi:10.1109/TBME.2022.3170025

10.	Cooper M a., Fletter P c., Zaszczurynski P j., Damaser M s. Comparison of air-charged and water-filled urodynamic pressure measurement catheters. Neurourology and Urodynamics. 2011;30: 329–334. doi:10.1002/nau.20991

11.	MAJERUS SJA, GARVERICK SL, SUSTER MA, FLETTER PC, DAMASER MS. Wireless, Ultra-Low-Power Implantable Sensor for Chronic Bladder Pressure Monitoring. ACM J Emerg Technol Comput Syst. 2012;8: 11. doi:10.1145/2180878.2180883

12.	Seo SG, Kim S, Yoo S, Oh S, Luan H, Lv Z, et al. Minimally Invasive, Bioadaptive Multimodal Sensor Probe with Safe Deployment for Real-Time Acute Compartment Syndrome Diagnosis. Advanced Science. 2025;12: e06942. doi:10.1002/advs.202506942
