# Supplementary material for: UROREC: A cystometry and electromyography recording system for analyzing urinary incontinence parameters in rat models
Source: PLoS One. 2026 Aug 26;21(8):e0356999. doi: 10.1371/journal.pone.0356999 (PMC13513990; doi:10.1371/journal.pone.0356999)
Supplement: Supplementary file 2 [file pone.0356999.s002.docx]

This manuscript presents a technically solid engineered system that addresses a real gap in preclinical urodynamic research. That said, while the engineering part is strong, the manuscript would benefit from clearer positioning,

Major Comments

1. The authors state that UROREC is “accessible, compact, and cost-effective,” but the manuscript lacks a direct comparison to existing systems, such as commercial or open-source systems.
2. What exactly differentiates UROREC from prior DAQ-based or microcontroller-based systems? Is the novelty primarily hardware integration, cost reduction, or signal quality? A table comparing cost, size, sampling rates, and number of channels would significantly strengthen the manuscript.
3. While the device is technically validated and tested in vivo, the biological validation is somewhat superficial.
4. The OAB model (acetic acid) is standard but low complexity. Suitable for initial validation, but represents an acute irritative model rather than a true neurogenic bladder. The observed changes are expected and may not fully reflect the device's sensitivity to chronic pathophysiology.
5. No comparison to a gold-standard system is presented, which is really important.
6. It is unclear whether the detected differences are device-sensitive or simply expected physiological changes.
7. Were recordings simultaneously obtained with a commercial system? How do absolute values such as MVP or PT compare to known benchmarks? Without this, it is difficult to assess true measurement accuracy vs internal consistency.
8. The study includes 15 rats, which is acceptable for a pilot but borderline for robust validation.
9. No power calculation is reported.
10. Multiple parameters are tested without correction for multiple comparisons
11. Some non-significant EMG parameters raise questions about sensitivity!
12. The authors should justify the sample size, clarify whether this is a proof-of-concept validation study
13. The electrical performance metrics are impressive; however, these are bench measurements, not in vivo. In vivo noise sources (movement, electrode variability) are not quantified. It would strengthen the manuscript to include the signal-to-noise ratio in vivo comparison of raw vs filtered signals failure rates or unusable recordings
14. Urethane anesthesia is an appropriate choice for preserving reflex micturition, but the manuscript should acknowledge its known effects on central modulation and sphincter coordination. Potential influence on EMG–pressure coupling is not discussed. Also, as a terminal anesthetic, it limits applicability to chronic or awake models.

Some questions:

Are PCB schematics, firmware, and code publicly available? Is assembly feasible for non-engineering labs? What is the actual cost breakdown? Without these, the claim of “accessible and scalable” is somewhat overstated.

Minor Comments

1. Consider moving some electronics detail to supplementary material.

2. Figures 7 and 8 (EMG + pressure traces) are informative but visually dense. Consider annotating key phases, such as filling, voiding, and burst onset, for clarity.

3. Some engineering terminology ( such as DRL circuit or DMA) is not explained for a biomedical audience. Add brief clarifications or references.

4. Abstract should be revised regarding key findings
