# Supplementary material for: UROREC: A cystometry and electromyography recording system for analyzing urinary incontinence parameters in rat models
Source: PLoS One. 2026 Aug 26;21(8):e0356999. doi: 10.1371/journal.pone.0356999 (PMC13513990; doi:10.1371/journal.pone.0356999)
Supplement: Supplementary file 3 [file pone.0356999.s003.docx]

April 14, 2026

Response to Editorial Office Requirements

Manuscript ID: PONE-D-26-17624

Dear Rochelle Ann Dabucol Librias,

We would like to express our gratitude for your preliminary evaluation and for the opportunity to address the technical requirements concerning animal welfare and humane endpoints for our manuscript. The manuscript has been revised, and the requested documentation has been furnished as follows:

1. Modifications to the Methods Section

a) Dedicated Sub-section. A new sub-section titled "Ethical Statement" has been added to the Materials and Methods. This section provides detailed information on animal care and uses in strict accordance with the ARRIVE 2.0 guidelines.
b) Humane Endpoints. We have explicitly defined the humane endpoints, including clinical signs such as abnormal posture, piloerection, and physiological instability.
c) Monitoring Frequency. The monitoring frequency has been clarified: animals were monitored every 30 minutes during the pre-experimental period and continuously throughout the surgical and recording stages.
d) Study Clarification. We have clarified in the text that this was an acute, non-survival experiment or terminal experiment; therefore, death was not a planned experimental endpoint.

2. Humane Endpoints Checklist

a) As requested, the PLOS ONE Humane Endpoints Checklist has been completed.
b) Following your specific instructions, this document has been uploaded to the system using the "Other" file type.

3. Experimental Duration and Anesthesia

a) The manuscript now specifies the total duration of the experiment, approximately 4 hours per subject.
b) We have provided detailed justification for the use of urethane as a terminal anesthetic agent due to its ability to preserve stable micturition reflexes during recording phases.

4. Data availability.

a) The minimal data set is available for peer review at <https://zenodo.org/records/19490037?token=eyJhbGciOiJIUzUxMiJ9.eyJpZCI6Ijg0YjBiODIzLTI3ODgtNDU5Mi05Y2RmLTA5ZDRlNDllZjI4OCIsImRhdGEiOnt9LCJyYW5kb20iOiIxMThlYjA1YmNiMzk1M2RkOTMwOGRlYzI4MGM2YWIzMCJ9.Z5J6ybvZgdn0xbPcajh7K2FBIR7FMqjw3p3JHPuobVMOYmrHsaJlEJ4-8OtKABN1DxZYxihjGzVCZVax_JYnOw>
b) The data will be made publicly available via DOI: <https://doi.org/10.5281/zenodo.19490037> upon formal acceptance of the manuscript.

We assert that these revisions comprehensively address the technical issues raised and ensure full compliance with PLOS ONE's editorial guidelines.

Yours sincerely,

Arturo Minor Martínez, PhD

Corresponding Author
