# Supplementary material for: UROREC: A cystometry and electromyography recording system for analyzing urinary incontinence parameters in rat models
Source: PLoS One. 2026 Aug 26;21(8):e0356999. doi: 10.1371/journal.pone.0356999 (PMC13513990; doi:10.1371/journal.pone.0356999)
Supplement: Supplementary file 4 [file pone.0356999.s004.docx]

**Response to the Academic Editor**

Dear Dr. Lee,

We sincerely thank you for coordinating the review process for our manuscript. We have carefully addressed all formatting, funding, and copyright requirements outlined in your letter. Below, we provide a point-by-point response detailing the adjustments implemented in this revised version.

**Journal Requirement 1:** Style and Formatting

Please ensure that your manuscript meets PLOS ONE's style requirements, including those for file naming.

**Response:** We have thoroughly reviewed the PLOS ONE style templates and updated the manuscript accordingly. Heading hierarchies, font layouts, and specific file naming conventions for the main text, figures, and supporting information files have been modified to comply strictly with the journal's guidelines.

**Journal Requirements 2 & 3:** Funding Statement and Acknowledgments

Funding information should not appear in the Acknowledgments section or other areas of your manuscript. Please remove any funding-related text from the manuscript and let us know how you would like to update your Funding Statement.

**Respons**e: We acknowledge this policy. We have completely removed all funding-related text from the Acknowledgments section of the manuscript. To replace our original default declaration, we request that our official Funding Statement online form be updated to reflect the following text (which has also been included in our amended Cover Letter):

"*Ramon Eduardo Cortina received a graduate maintenance grant from the Secretariat of Science, Humanities, Technology, and Innovation (SECIHTI), Mexico. Material support in the form of laboratory animals and facility access was provided by the Center for Research and Advanced Studies (Cinvestav), Zacatenco Unit, through the Laboratory Animal Production and Experimentation Unit (UPEAL). The funders had no role in study design, data collection and analysis, decision to publish, or preparation of the manuscript.*"

**Journal Requirement 4:** Data Availability Statement

Please note that, though access restrictions are acceptable now, your entire data will need to be made freely accessible if your manuscript is accepted for publication.

**Response:** We fully adhere to PLOS ONE’s open data policy. All primary data tracks, code, firmware, and hardware design files (schematics, layouts, and Gerbers) necessary to replicate our results have been permanently deposited in a public repository via Zenodo (<https://doi.org/10.5281/zenodo.19490036> ). This active repository link is freely accessible and has been integrated into the Data Availability Statement of the manuscript.

**Journal Requirement 5:** Ethics Statement Position

Your ethics statement should only appear in the Methods section of your manuscript. If your ethics statement is written in any section besides the Methods, please delete it from any other section.

**Response:** We have verified compliance with this requirement. The institutional animal ethics approval statement is located exclusively within the Materials and Methods section. It does not appear in any other section of the manuscript.

**Journal Requirement 6:** Copyright and Figure Permissions (Figures 3, 4, and 5, old version)

We note that Figures 3, 4, and 5 in your submission contains images which may be copyrighted...

**Response**: We would like to clarify that Figures 3, 4, and 5 from the original submission (which correspond to Figure 2, S3 Fig in S1 File, and Figure 3 in this revised version, respectively) are entirely original works created from scratch by the authors specifically for this study. No third-party assets, copyrighted clipart, or restricted digital images were utilized in these panels. Therefore, these figures do not infringe upon any existing copyrights and are fully eligible for publication.

**Response to the Reviewers**

We would like to express our sincere gratitude to both reviewers for their time, dedication, and highly constructive feedback. Their insightful comments and valuable suggestions have significantly helped us improve the clarity, technical rigor, and overall quality of our manuscript. We have carefully addressed all the points raised during the review process. Below, we provide a point-by-point response to each reviewer's comments, along with the details of the corresponding modifications implemented in the revised manuscript.

**Reviewer 1**

Major Comments

1. **Reviewer Comment:** The authors state that UROREC is “accessible, compact, and cost-effective,” but the manuscript lacks a direct comparison to existing systems, such as commercial or open-source systems.

**Response:** We agree that a direct comparison is essential to substantiate these claims. Following the reviewer's suggestion, we have included a comprehensive benchmarking framework in a new Table 4 within the Discussion section (Lines 387–389). This table directly compares UROREC against market-leading commercial workstations (BIOPAC and AD Instruments) and open-source platforms (OpenBCI) across key metrics such as cost, isolation, sampling rate, and portability.

2. **Reviewer Comment:** What exactly differentiates UROREC from prior DAQ-based or microcontroller-based systems? Is the novelty primarily hardware integration, cost reduction, or signal quality? A table comparing cost, size, sampling rates, and number of channels would significantly strengthen the manuscript.

**Response:** We appreciate this observation. The primary novelty of UROREC lies in cost reduction and custom hardware integration specifically optimized for preclinical urodynamics. As explicitly outlined in the Introduction (Lines 86–99), the platform does not intend to replace high-end commercial workstations, but rather to eliminate logistical and financial barriers by providing an accessible, compact, and reliable single-board alternative. These structural differences, along with comparisons of cost, size, sampling rates, and channels, are now systematically detailed in Table 4 (Discussion, Lines 387–389) as requested.

3. **Reviewer Comment:** While the device is technically validated and tested in vivo, the biological validation is somewhat superficial.

**Response:** We appreciate this perspective. While the study's scope focuses on technical architecture rather than exploring novel biological mechanisms, the in vivo validation was designed to rigorously test the system against complex physiological dynamics. Specifically, the platform successfully recorded microvolt-range EUS oscillations, high-frequency bursting, and exact electromyography-pressure coupling during active voiding, as reported in Table 3 and addressed in the Discussion section. This precise replication of established, intricate urological phenomena reported in the literature provides a robust biological benchmark to validate our results.

4. **Reviewer Comment:** The OAB model (acetic acid) is standard but low complexity. Suitable for initial validation, but represents an acute irritative model rather than a true neurogenic bladder. The observed changes are expected and may not fully reflect the device's sensitivity to chronic pathophysiology.

**Response:** We entirely agree with this distinction. The acute chemical irritation model was selected strictly as a predictable baseline setup for initial hardware validation. We acknowledge that evaluating the platform under chronic or neurogenic conditions is a necessary next step. To ensure absolute clarity and prevent any misrepresentation, we have thoroughly revised the manuscript as follows:

- Scope clarification: Explicitly framed the in vivo validation as a baseline proof-of-concept in the Abstract (Lines 25–27) and Introduction (Lines 92–93).
- Terminology correction: Updated the subsection header (Line 185) and Figure 4 caption (Lines 308–312) to correctly state "Overactive Bladder" instead of "neurogenic bladder".
- Limitations expanded: Revised the Discussion (Lines 433–439) to acknowledge this pilot validation as the foundational step required to plan future chronic trials.

5. **Reviewer Comment:** No comparison to a gold-standard system is presented, which is really important.

**Response:** We acknowledge that a simultaneous, side-by-side parallel recording with a commercial system was not performed in this initial pilot phase. However, to address the critical distinction between true measurement accuracy and internal consistency, UROREC’s absolute baseline values were rigorously benchmarked against established historical metrics from high-end commercial workstations. As detailed in the Discussion (Lines 357–368), our recorded baseline Maximum Voiding Pressure and Pressure Threshold fall strictly within the classic physiological ranges reported by gold-standard urodynamic studies. To ensure full transparency, we have explicitly added this lack of simultaneous cross-validation as a limitation in the Discussion section (Lines 439-444), framing it as a key objective for future experimental trials.

6. **Reviewer Comment:** It is unclear whether the detected differences are device-sensitive or simply expected physiological changes.

**Response:** We appreciate this distinction. The differences detected are indeed the expected physiological alterations induced by the acute OAB model. However, UROREC’s technical sensitivity is demonstrated precisely by its high parameter-specificity and resolution. As shown in Table 3 and detailed in the Discussion (Lines 415–421), the platform does not record an arbitrary or uniform shift across all variables; instead, it selectively captures statistically significant changes in afferent-mediated parameters (such as ICI and PT) while faithfully reflecting the stability of efferent neuromuscular parameters (such as Phasic RMS, AUC, and Duration). This high level of discrimination confirms that the system possesses the necessary sensitivity to track true pathophysiological dynamics without instrument-induced distortions.

7. **Reviewer Comment:** Were recordings simultaneously obtained with a commercial system? How do absolute values such as MVP or PT compare to known benchmarks? Without this, it is difficult to assess true measurement accuracy vs internal consistency.

**Response:** Simultaneous parallel recordings were not obtained during this initial pilot stage. However, to directly resolve the question of true measurement accuracy versus internal consistency, we explicitly benchmarked UROREC's absolute quantified values against established gold-standard benchmarks within the Discussion section (Lines 357–368). As detailed in the text, our baseline Maximum Voiding Pressure (MVP = 32.29 cmH2O) and Pressure Threshold (PT = 7.42 cmH2O) fall strictly within the classic physiological ranges reported for urethane-anesthetized female Wistar rats (20–35 cmH2O for MVP and 5–10 cmH2O for PT). This precise numerical correlation validates that the platform accurately measures absolute physical parameters rather than merely tracking internal relative trends.

8. **Reviewer Comment:** The study includes 15 rats, which is acceptable for a pilot but borderline for robust validation.

**Response:** We agree with the reviewer's assessment regarding the cohort size for a full biological study. This work was intentionally designed as a paired pilot validation and engineering proof-of-concept under institutional ethical minimization guidelines. Following the reviewer's feedback, we have explicitly clarified this scope throughout the manuscript, including the Abstract (Line 26), Introduction (Line 87), and Statistical Analysis (Line 277). Furthermore, as explained in the Discussion (Lines 444–449), a primary methodological goal of this 15-subject pilot cohort was to generate the preliminary statistical baseline required to perform accurate a priori power calculations for future large-scale trials.

9. **Reviewer Comment:** No power calculation is reported.

**Response:** We acknowledge that a formal a priori statistical power calculation was not performed due to the technical pilot validation and proof-of-concept nature of this hardware study. To ensure full methodological transparency, we have explicitly stated this constraint in the Statistical Analysis section (Lines 276–279), clarifying that the sample size was determined based on institutional ethical minimization guidelines and established pilot benchmarks in the literature.

10. **Reviewer Comment:** Multiple parameters are tested without correction for multiple comparisons

**Response:** We fully agree that evaluating multiple urodynamic metrics increases the risk of Type I errors and appreciate this recommendation to enhance statistical rigor. To address this, we have revised our analysis and applied the Benjamini-Hochberg False Discovery Rate (FDR) procedure across all 18 evaluated cystometric and electromyographic parameters. Crucially, due to the robustness of the biological changes captured, all 12 previously identified significant parameters successfully maintained their statistical significance (p < 0.05) after the FDR adjustment. These updates have been transparently integrated into the Statistical Analysis section (Lines 273–276) and the footnote of Table 3 (Lines 333–336).

11. **Reviewer Comment:** Some non-significant EMG parameters raise questions about sensitivity!

**Response:** We appreciate this observation. The non-significant outcomes in specific EUS-EMG parameters (Phasic RMS, Phasic AUC, and Phasic Duration) reflect genuine neurobiological behavior rather than a lack of hardware sensitivity Discussion (Lines 357–365). The acute acetic acid model induces detrusor hyperactivity via afferent pathway irritation while leaving the efferent neuromuscular integrity of the external urethral sphincter (EUS) intact as detailed in the Discussion (Lines 415–422) and visualized in Figures 4 and 5.

12. **Reviewer Comment:** The authors should justify the sample size, clarify whether this is a proof-of-concept validation study

**Response:** We have explicitly revised the manuscript to clarify that this work is a technical proof-of-concept and pilot validation study across the Abstract (Line 26), Introduction (Lines 86–90), Statistical Analysis section (Lines 276–279), and Discussion (Lines 444–449). Regarding the sample size, the use of N = 15 subjects is methodologically justified by our strictly paired experimental design, where each animal served as its own baseline control before and after the acute challenge. This framework eliminates inter-animal biological variability from the error variance, significantly increasing statistical efficiency and data precision while strictly adhering to the ethical "Reduction" principle of animal research guidelines.

13. **Reviewer Comment:** The electrical performance metrics are impressive; however, these are bench measurements, not in vivo. In vivo noise sources (movement, electrode variability) are not quantified. It would strengthen the manuscript to include the signal-to-noise ratio in vivo comparison of raw vs filtered signals failure rates or unusable recordings

**Response:** We entirely agree that quantifying in vivo noise and operational reliability significantly strengthens the technical validation of the platform under real experimental conditions. Following the reviewer's suggestion, we have updated the manuscript to include these metrics. These quantifications have been integrated into Table 2 (Lines 287) and explicitly detailed in the *In vivo* Measurements section (Lines 303–307).

14. **Reviewer Comment:** Urethane anesthesia is an appropriate choice for preserving reflex micturition, but the manuscript should acknowledge its known effects on central modulation and sphincter coordination. Potential influence on EMG–pressure coupling is not discussed. Also, as a terminal anesthetic, it limits applicability to chronic or awake models

**Response:** We agree with this methodological perspective. While urethane is the standard for acute urodynamics due to reflex preservation, it is well-documented to depress supraspinal central modulation, which can influence fine EUS coordination and EMG–pressure coupling. Furthermore, its terminal classification inherently constrains the direct translation of these acute data to awake or chronic setups. To address this transparently, we have expanded the limitations section in the Discussion (Lines 403-407), explicitly addressing these anesthetic implications on sphincter dynamics and framing the transition to non-terminal, awake monitoring configurations as a primary objective for future iterations of the platform (Lines 433-437).

**Reviewer General Questions**

**Reviewer 1:** Are PCB schematics, firmware, and code publicly available? Is assembly feasible for non-engineering labs? What is the actual cost breakdown? Without these, the claim of “accessible and scalable” is somewhat overstated.

**Response:** We fully agree that true accessibility requires absolute transparency and clear, documented replication pathways within the manuscript.

- Public Availability: All hardware design files (schematics, PCB layouts, Gerbers) and firmware (C/C++) are permanently available via Zenodo: https://doi.org/10.5281/zenodo.19490036. This repository link has been formally added to the Data Availability Statement (Lines 475–478).
- Assembly Feasibility (Replication Pathways): To ensure non-engineering laboratories can easily adopt UROREC, we have integrated a detailed breakdown of the two replication workflows (Commercial PCBA vs. In-house DIY) into the Discussion section (Lines 450–458, and Supplementary Material, S1 File).
- Cost Breakdown: A fully itemized, component-by-component cost breakdown has been added as Supplementary Material (S1 File) and is now explicitly cited in the main text (Discussion, Line 450-451).

**Minor Comment**

**Minor Comment 1:** Consider moving some electronics detail to supplementary material.

**Response**: We have followed this suggestion to improve the manuscript's readability for a broader audience. Detailed hardware schematics and components (Electromyography acquisition circuit Line 100, Cystometry acquisition circuit Line 124 and Digital processing and interface unit Line 138 sections) have been relocated to the Supplementary Material (S1 File). The main text now retains only the essential architectural blocks and functional descriptions.

**Minor Comment 2:** Figures 7 and 8 (EMG + pressure traces) are informative but visually dense. Consider annotating key phases, such as filling, voiding, and burst onset, for clarity.

**Response**: We agree that these plots benefit from visual demarcation to improve readability. We have updated these figures, which correspond to Figure 4 and Figure 5 in the revised manuscript, by adding explicit text labels and arrows directly onto the traces. These annotations now clearly highlight the Filling Phase, Burst Onset, and Voiding Phase, replacing the originally dense plots with high-resolution, physiologically mapped versions that are much easier to interpret.

**Minor Comment 3:** Some engineering terminology (such as DRL circuit or DMA) is not explained for a biomedical audience. Add brief clarifications or references.

**Response**: We appreciate this feedback. To ensure clarity for the biomedical community, we have added brief, accessible definitions for these technical mechanisms. Driven Right Leg (DRL, Lines 118-121). Direct Memory Access (DMA, Lines 144-146).

**Minor Comment 4:** Abstract should be revised regarding key findings.

**Response**: We agree with this recommendation. The Abstract has been thoroughly revised to replace purely qualitative statements with our key quantitative and statistical findings. Specifically, it now explicitly reports the precise physiological shifts captured by UROREC during this pilot validation.

**Reviewer 2**

**General Comments and Evaluation**

1. **Reviewer 2 Comment (Introduction):** Clear and concise introduction to the problem and current limitations. The objectives are clearly established.

**Response**: We sincerely thank the reviewer for this positive evaluation. We are glad to hear that the introduction successfully provides a clear, concise contextualization of the problem and its current limitations.

2. **Reviewer 2 Comment (Methods):** The Methods section is very lengthy, and the subheaders make it somewhat hard to follow. Given PLOS ONE’s broad multidisciplinary readership, some of the more technical and mathematical aspects of the model could perhaps be moved to the supplementary section, while preserving enough detail in the main text for readers to understand the system’s validation and reproducibility.

**Response:** We agree that streamlining the highly technical engineering blocks enhances the manuscript's readability for PLOS ONE’s broad multidisciplinary audience. Following this recommendation, we have thoroughly reorganized and condensed the Materials and Methods section. Component-level routing matrices, electronic layout strategies, and secondary mathematical circuit equations have been relocated to the Supplementary Material (S1 File).

3. **Reviewer 2 Comment (Results):** There are several figures and tables, and some could probably be moved to supplemental information. Otherwise, the results are well presented and specifically show the differences between healthy controls and the OAB model, including the expected changes that have been reported in the literature.

**Response:** We appreciate the reviewer's positive evaluation of our physiological findings. To prevent visual oversaturation and streamline the Results section, we have carefully re-evaluated our display items. Specifically, we have relocated the benchtop technical characterization plots and the raw hardware verification data to the Supplementary Material (S1 File).

4. **Reviewer 2 Comment (Discussion):** The authors clearly explain the results, how they compare with the literature, and how the UROREC system is advantageous by measuring CMG and EUS-EMG simultaneously, allowing both signals to validate each other in real time. The limitations are also addressed comprehensively, including the use of anesthesia and its potential effects on micturition patterns, as well as the significant decrease in the 1st PD in the pathological model. For readers less familiar with the costs and current devices used in other laboratories, it would be useful to dedicate more space to explaining why UROREC is particularly attainable and how it could help scale up these investigations.

**Response:** We appreciate these positive remarks regarding our discussion and limitations framework. To provide a clearer context for readers unfamiliar with the high financial barriers of conventional urodynamic workstations, we have expanded the Discussion section (Lines 387–457).
