# Supplementary material for: UROREC: A cystometry and electromyography recording system for analyzing urinary incontinence parameters in rat models
Source: PLoS One. 2026 Aug 26;21(8):e0356999. doi: 10.1371/journal.pone.0356999 (PMC13513990; doi:10.1371/journal.pone.0356999)
Supplement: Peer review history

# Author comment: Author response to Decision Letter 0

**DOI:** 10.1371/journal.pone.0356999.r001

14 Apr 2026

Attachment Submitted filename: Response to Reviewers_1.docx

# Peer review: Decision Letter 0

**DOI:** 10.1371/journal.pone.0356999.r002

**Authors:** Richard Lee

2 Jun 2026

Please submit your revised manuscript by Jul 17 2026 11:59PM. If you will need more time than this to complete your revisions, please reply to this message or contact the journal office at plosone@plos.org. When you're ready to submit your revision, log on to https://www.editorialmanager.com/pone/ and select the 'Submissions Needing Revision' folder to locate your manuscript file.

- A letter that responds to each point raised by the academic editor and reviewer(s). You should upload this letter as a separate file labeled 'Response to Reviewers'.
- A marked-up copy of your manuscript that highlights changes made to the original version. You should upload this as a separate file labeled 'Revised Manuscript with Track Changes'.
- An unmarked version of your revised paper without tracked changes. You should upload this as a separate file labeled 'Manuscript'.

If you would like to make changes to your financial disclosure, please include your updated statement in your cover letter. Guidelines for resubmitting your figure files are available below the reviewer comments at the end of this letter.

If applicable, we recommend that you deposit your laboratory protocols in protocols.io to enhance the reproducibility of your results. Protocols.io assigns your protocol its own identifier (DOI) so that it can be cited independently in the future. For instructions see: https://journals.plos.org/plosone/s/submission-guidelines#loc-laboratory-protocols. Additionally, PLOS ONE offers an option for publishing peer-reviewed Lab Protocol articles, which describe protocols hosted on protocols.io. Read more information on sharing protocols at https://plos.org/protocols?utm_medium=editorial-email&utm_source=authorletters&utm_campaign=protocols.

As the corresponding author, your ORCID iD is verified in the submission system and will appear in the published article. PLOS supports the use of ORCID, and we encourage all coauthors to register for an ORCID iD and use it as well. Please encourage your coauthors to verify their ORCID iD within the submission system before final acceptance, as unverified ORCID iDs will not appear in the published article. *Only*  the individual author can complete the verification step; PLOS staff *cannot*  verify ORCID iDs on behalf of authors.

We look forward to receiving your revised manuscript.

Kind regards,

Richard Kao Lee, M.D.

Academic Editor

PLOS One

Journal Requirements:

When submitting your revision, we need you to address these additional requirements.

1. Please ensure that your manuscript meets PLOS ONE's style requirements, including those for file naming. The PLOS ONE style templates can be found at https://journals.plos.org/plosone/s/file?id=wjVg/PLOSOne_formatting_sample_main_body.pdf and https://journals.plos.org/plosone/s/file?id=ba62/PLOSOne_formatting_sample_title_authors_affiliations.pdf

2. Thank you for stating the following financial disclosure:

[The author(s) received no specific funding for this work.].

At this time, please address the following queries:

a) Please clarify the sources of funding (financial or material support) for your study. List the grants or organizations that supported your study, including funding received from your institution.

b) State what role the funders took in the study. If the funders had no role in your study, please state: “The funders had no role in study design, data collection and analysis, decision to publish, or preparation of the manuscript.”

c) If any authors received a salary from any of your funders, please state which authors and which funders.

d) If you did not receive any funding for this study, please state: “The authors received no specific funding for this work.”

Please include your amended statements within your cover letter; we will change the online submission form on your behalf.

3. Thank you for stating the following in your manuscript:

[Ramon Eduardo Cortina received a maintenance grant from the Secretariat of Science, Humanities, Technology, and Innovation (SECIHTI), Mexico. The Center for Research and Advanced Studies, Zacatenco Unit, supported this work through the Laboratory Animal Production and Experimentation Unit (UPEAL).]

We note that you have provided funding information that is not currently declared in your Funding Statement. However, funding information should not appear in the Acknowledgments section or other areas of your manuscript. We will only publish funding information present in the Funding Statement section of the online submission form.

Please remove any funding-related text from the manuscript and let us know how you would like to update your Funding Statement. Currently, your Funding Statement reads as follows:

[The author(s) received no specific funding for this work.]

Please include your amended statements within your cover letter; we will change the online submission form on your behalf.

4. When completing the data availability statement of the submission form, you indicated that you will make your data available on acceptance. We strongly recommend all authors decide on a data sharing plan before acceptance, as the process can be lengthy and hold up publication timelines. Please note that, though access restrictions are acceptable now, your entire data will need to be made freely accessible if your manuscript is accepted for publication. This policy applies to all data except where public deposition would breach compliance with the protocol approved by your research ethics board. If you are unable to adhere to our open data policy, please kindly revise your statement to explain your reasoning and we will seek the editor's input on an exemption. Please be assured that, once you have provided your new statement, the assessment of your exemption will not hold up the peer review process.

5. Your ethics statement should only appear in the Methods section of your manuscript. If your ethics statement is written in any section besides the Methods, please delete it from any other section.

6. We note that Figures 3, 4, and 5 in your submission contains images which may be copyrighted. All PLOS content is published under the Creative Commons Attribution License (CC BY 4.0), which means that the manuscript, images, and Supporting Information files will be freely available online, and any third party is permitted to access, download, copy, distribute, and use these materials in any way, even commercially, with proper attribution. For more information, see our copyright guidelines: http://journals.plos.org/plosone/s/licenses-and-copyright.

We require you to either (1) present written permission from the copyright holder to publish these figures specifically under the CC BY 4.0 license, or (2) remove the figures from your submission:

1. You may seek permission from the original copyright holder of Figures 3, 4, and 5 to publish the content specifically under the CC BY 4.0 license.

We recommend that you contact the original copyright holder with the Content Permission Form (http://journals.plos.org/plosone/s/file?id=7c09/content-permission-form.pdf) and the following text:

“I request permission for the open-access journal PLOS ONE to publish XXX under the Creative Commons Attribution License (CCAL) CC BY 4.0 (http://creativecommons.org/licenses/by/4.0/). Please be aware that this license allows unrestricted use and distribution, even commercially, by third parties. Please reply and provide explicit written permission to publish XXX under a CC BY license and complete the attached form.”

Please upload the completed Content Permission Form or other proof of granted permissions as an "Other" file with your submission.

In the figure caption of the copyrighted figure, please include the following text: “Reprinted from [ref] under a CC BY license, with permission from [name of publisher], original copyright [original copyright year].”

2. If you are unable to obtain permission from the original copyright holder to publish these figures under the CC BY 4.0 license or if the copyright holder’s requirements are incompatible with the CC BY 4.0 license, please either i) remove the figure or ii) supply a replacement figure that complies with the CC BY 4.0 license. Please check copyright information on all replacement figures and update the figure caption with source information. If applicable, please specify in the figure caption text when a figure is similar but not identical to the original image and is therefore for illustrative purposes only.

If the reviewer comments include a recommendation to cite specific previously published works, please review and evaluate these publications to determine whether they are relevant and should be cited. There is no requirement to cite these works unless the editor has indicated otherwise.

[Note: HTML markup is below. Please do not edit.]

Reviewers' comments:

Reviewer's Responses to Questions

**Comments to the Author**

1. Is the manuscript technically sound, and do the data support the conclusions?

Reviewer #1: Yes

Reviewer #2: Yes

**********

2. Has the statistical analysis been performed appropriately and rigorously?

Reviewer #1: Yes

Reviewer #2: Yes

**********

3. Have the authors made all data underlying the findings in their manuscript fully available?

The PLOS Data policy

Reviewer #1: No

Reviewer #2: Yes

**********

4. Is the manuscript presented in an intelligible fashion and written in standard English?

Reviewer #1: Yes

Reviewer #2: Yes

**********

Reviewer #1: This manuscript presents a technically solid engineered system that addresses a real gap in preclinical urodynamic research. That said, while the engineering part is strong, the manuscript would benefit from clearer positioning (see all comments below)

Reviewer #2: This study aims to present and evaluate the UROREC system, a combined platform to measure concurrent CMG and EUS-EMG activity in murine models.

Introduction: Clear and concise introduction to the problem and current limitations. The objectives are clearly established.

Methods: The Methods section is very lengthy, and the subheaders make it somewhat hard to follow. Given PLOS ONE’s broad multidisciplinary readership, some of the more technical and mathematical aspects of the model could perhaps be moved to the supplementary section, while preserving enough detail in the main text for readers to understand the system’s validation and reproducibility. Otherwise, the animal model used is a known and widely used model for OAB, with specific details on the surgical procedure and anesthetic used, which is important given that anesthesia is known to affect voiding patterns. Bladder filling rates and normalization procedures are also well explained. There is an adequate statistical analysis section.

Results: There are several figures and tables, and some could probably be moved to the supplemental information. Otherwise, the results are well presented and specifically show the differences between healthy controls and the OAB model, including the expected changes that have been reported in the literature.

Discussion: The authors clearly explain the results, how they compare with the literature, and how the UROREC system is advantageous by measuring CMG and EUS-EMG simultaneously, allowing both signals to validate each other in real time. The limitations are also addressed comprehensively, including the use of anesthesia and its potential effects on micturition patterns, as well as the significant decrease in the 1st PD in the pathological model. For readers less familiar with the costs and current devices used in other laboratories, it would be useful to dedicate more space to explaining why UROREC is particularly attainable and how it could help scale up these investigations.

I congratulate the authors on performing a comprehensive analysis of their model, which appears to measure important urodynamic parameters effectively and concomitantly in a more attainable manner. They demonstrate that their system can reliably reproduce expected findings in both healthy and pathological OAB bladders.

**********

what does this mean?). If published, this will include your full peer review and any attached files.

If you choose “no”, your identity will remain anonymous but your review may still be made public.

**Do you want your identity to be public for this peer review?**  For information about this choice, including consent withdrawal, please see our Privacy Policy

Reviewer #1: No

Reviewer #2: No

**********

[NOTE: If reviewer comments were submitted as an attachment file, they will be attached to this email and accessible via the submission site. Please log into your account, locate the manuscript record, and check for the action link "View Attachments". If this link does not appear, there are no attachment files.]

To ensure your figures meet our technical requirements, please review our figure guidelines: https://journals.plos.org/plosone/s/figures

You may also use PLOS’s free figure tool, NAAS, to help you prepare publication quality figures: https://journals.plos.org/plosone/s/figures#loc-tools-for-figure-preparation.

NAAS will assess whether your figures meet our technical requirements by comparing each figure against our figure specifications.

Attachment Submitted filename: Reviewer comments.docx

# Author comment: Author response to Decision Letter 1

**DOI:** 10.1371/journal.pone.0356999.r003

26 Jun 2026

Response to the Academic Editor

Dear Dr. Lee,

We sincerely thank you for coordinating the review process for our manuscript. We have carefully addressed all formatting, funding, and copyright requirements outlined in your letter. Below, we provide a point-by-point response detailing the adjustments implemented in this revised version.

Journal Requirement 1: Style and Formatting

Please ensure that your manuscript meets PLOS ONE's style requirements, including those for file naming.

Response: We have thoroughly reviewed the PLOS ONE style templates and updated the manuscript accordingly. Heading hierarchies, font layouts, and specific file naming conventions for the main text, figures, and supporting information files have been modified to comply strictly with the journal's guidelines.

Journal Requirements 2 & 3: Funding Statement and Acknowledgments

Funding information should not appear in the Acknowledgments section or other areas of your manuscript. Please remove any funding-related text from the manuscript and let us know how you would like to update your Funding Statement.

Response: We acknowledge this policy. We have completely removed all funding-related text from the Acknowledgments section of the manuscript. To replace our original default declaration, we request that our official Funding Statement online form be updated to reflect the following text (which has also been included in our amended Cover Letter):

"Ramon Eduardo Cortina received a graduate maintenance grant from the Secretariat of Science, Humanities, Technology, and Innovation (SECIHTI), Mexico. Material support in the form of laboratory animals and facility access was provided by the Center for Research and Advanced Studies (Cinvestav), Zacatenco Unit, through the Laboratory Animal Production and Experimentation Unit (UPEAL). The funders had no role in study design, data collection and analysis, decision to publish, or preparation of the manuscript."

Journal Requirement 4: Data Availability Statement

Please note that, though access restrictions are acceptable now, your entire data will need to be made freely accessible if your manuscript is accepted for publication.

Response: We fully adhere to PLOS ONE’s open data policy. All primary data tracks, code, firmware, and hardware design files (schematics, layouts, and Gerbers) necessary to replicate our results have been permanently deposited in a public repository via Zenodo (https://doi.org/10.5281/zenodo.19490036). This active repository link is freely accessible and has been integrated into the Data Availability Statement of the manuscript.

Journal Requirement 5: Ethics Statement Position

Your ethics statement should only appear in the Methods section of your manuscript. If your ethics statement is written in any section besides the Methods, please delete it from any other section.

Response: We have verified compliance with this requirement. The institutional animal ethics approval statement is located exclusively within the Materials and Methods section. It does not appear in any other section of the manuscript.

Journal Requirement 6: Copyright and Figure Permissions (Figures 3, 4, and 5, old version)

We note that Figures 3, 4, and 5 in your submission contains images which may be copyrighted...

Response: We would like to clarify that Figures 3, 4, and 5 from the original submission (which correspond to Figure 2, S3 Fig in S1 File, and Figure 3 in this revised version, respectively) are entirely original works created from scratch by the authors specifically for this study. No third-party assets, copyrighted clipart, or restricted digital images were utilized in these panels. Therefore, these figures do not infringe upon any existing copyrights and are fully eligible for publication.

Response to the Reviewers

We would like to express our sincere gratitude to both reviewers for their time, dedication, and highly constructive feedback. Their insightful comments and valuable suggestions have significantly helped us improve the clarity, technical rigor, and overall quality of our manuscript. We have carefully addressed all the points raised during the review process. Below, we provide a point-by-point response to each reviewer's comments, along with the details of the corresponding modifications implemented in the revised manuscript.

Reviewer 1

Major Comments

1. Reviewer Comment: The authors state that UROREC is “accessible, compact, and cost-effective,” but the manuscript lacks a direct comparison to existing systems, such as commercial or open-source systems.

Response: We agree that a direct comparison is essential to substantiate these claims. Following the reviewer's suggestion, we have included a comprehensive benchmarking framework in a new Table 4 within the Discussion section (Lines 387–389). This table directly compares UROREC against market-leading commercial workstations (BIOPAC and AD Instruments) and open-source platforms (OpenBCI) across key metrics such as cost, isolation, sampling rate, and portability.

2. Reviewer Comment: What exactly differentiates UROREC from prior DAQ-based or microcontroller-based systems? Is the novelty primarily hardware integration, cost reduction, or signal quality? A table comparing cost, size, sampling rates, and number of channels would significantly strengthen the manuscript.

Response: We appreciate this observation. The primary novelty of UROREC lies in cost reduction and custom hardware integration specifically optimized for preclinical urodynamics. As explicitly outlined in the Introduction (Lines 86–99), the platform does not intend to replace high-end commercial workstations, but rather to eliminate logistical and financial barriers by providing an accessible, compact, and reliable single-board alternative. These structural differences, along with comparisons of cost, size, sampling rates, and channels, are now systematically detailed in Table 4 (Discussion, Lines 387–389) as requested.

3. Reviewer Comment: While the device is technically validated and tested in vivo, the biological validation is somewhat superficial.

Response: We appreciate this perspective. While the study's scope focuses on technical architecture rather than exploring novel biological mechanisms, the in vivo validation was designed to rigorously test the system against complex physiological dynamics. Specifically, the platform successfully recorded microvolt-range EUS oscillations, high-frequency bursting, and exact electromyography-pressure coupling during active voiding, as reported in Table 3 and addressed in the Discussion section. This precise replication of established, intricate urological phenomena reported in the literature provides a robust biological benchmark to validate our results.

4. Reviewer Comment: The OAB model (acetic acid) is standard but low complexity. Suitable for initial validation, but represents an acute irritative model rather than a true neurogenic bladder. The observed changes are expected and may not fully reflect the device's sensitivity to chronic pathophysiology.

Response: We entirely agree with this distinction. The acute chemical irritation model was selected strictly as a predictable baseline setup for initial hardware validation. We acknowledge that evaluating the platform under chronic or neurogenic conditions is a necessary next step. To ensure absolute clarity and prevent any misrepresentation, we have thoroughly revised the manuscript as follows:

• Scope clarification: Explicitly framed the in vivo validation as a baseline proof-of-concept in the Abstract (Lines 25–27) and Introduction (Lines 92–93).

• Terminology correction: Updated the subsection header (Line 185) and Figure 4 caption (Lines 308–312) to correctly state "Overactive Bladder" instead of "neurogenic bladder".

• Limitations expanded: Revised the Discussion (Lines 433–439) to acknowledge this pilot validation as the foundational step required to plan future chronic trials.

5. Reviewer Comment: No comparison to a gold-standard system is presented, which is really important.

Response: We acknowledge that a simultaneous, side-by-side parallel recording with a commercial system was not performed in this initial pilot phase. However, to address the critical distinction between true measurement accuracy and internal consistency, UROREC’s absolute baseline values were rigorously benchmarked against established historical metrics from high-end commercial workstations. As detailed in the Discussion (Lines 357–368), our recorded baseline Maximum Voiding Pressure and Pressure Threshold fall strictly within the classic physiological ranges reported by gold-standard urodynamic studies. To ensure full transparency, we have explicitly added this lack of simultaneous cross-validation as a limitation in the Discussion section (Lines 439-444), framing it as a key objective for future experimental trials.

6. Reviewer Comment: It is unclear whether the detected differences are device-sensitive or simply expected physiological changes.

Response: We appreciate this distinction. The differences detected are indeed the expected physiological alterations induced by the acute OAB model. However, UROREC’s technical sensitivity is demonstrated precisely by its high parameter-specificity and resolution. As shown in Table 3 and detailed in the Discussion (Lines 415–421), the platform does not record an arbitrary or uniform shift across all variables; instead, it selectively captures statistically significant changes in afferent-mediated parameters (such as ICI and PT) while faithfully reflecting the stability of efferent neuromuscular parameters (such as Phasic RMS, AUC, and Duration). This high level of discrimination confirms that the system possesses the necessary sensitivity to track true pathophysiological dynamics without instrument-induced distortions.

7. Reviewer Comment: Were recordings simultaneously obtained with a commercial system? How do absolute values such as MVP or PT compare to known benchmarks? Without this, it is difficult to assess true measurement accuracy vs internal consistency.

Response: Simultaneous parallel recordings were not obtained during this initial pilot stage. However, to directly resolve the question of true measurement accuracy versus internal consistency, we explicitly benchmarked UROREC's absolute quantified values against established gold-standard benchmarks within the Discussion section (Lines 357–368). As detailed in the text, our baseline Maximum Voiding Pressure (MVP = 32.29 cmH2O) and Pressure Threshold (PT = 7.42 cmH2O) fall strictly within the classic physiological ranges reported for urethane-anesthetized female Wistar rats (20–35 cmH2O for MVP and 5–10 cmH2O for PT). This precise numerical correlation validates that the platform accurately measures absolute physical parameters rather than merely tracking internal relative trends.

8. Reviewer Comment: The study includes 15 rats, which is acceptable for a pilot but borderline for robust validation.

Response: We agree with the reviewer's assessment regarding the cohort size for a full biological study. This work was intentionally designed as a paired pilot validation and engineering proof-of-concept under institutional ethical minimization guidelines. Following the reviewer's feedback, we have explicitly clarified this scope throughout the manuscript, including the Abstract (Line 26), Introduction (Line 87), and Statistical Analysis (Line 277). Furthermore, as explained in the Discussion (Lines 444–449), a primary methodological goal of this 15-subject pilot cohort was to generate the preliminary statistical baseline required to perform accurate a priori power calculations for future large-scale trials.

9. Reviewer Comment: No power calculation is reported.

Response: We acknowledge that a formal a priori statistical power calculation was not performed due to the technical pilot validation and proof-of-concept nature of this hardware study. To ensure full methodological transparency, we have explicitly stated this constraint in the Statistical Analysis section (Lines 276–279), clarifying that the sample size was determined based on institutional ethical minimization guidelines and established pilot benchmarks in the literature.

10. Reviewer Comment: Multiple parameters are tested without correction for multiple comparisons

Response: We fully agree that evaluating multiple urodynamic metrics increases the risk of Type I errors and appreciate this recommendation to enhance statistical rigor. To address this, we have revised our analysis and applied the Benjamini-Hochberg False Discovery Rate (FDR) procedure across all 18 evaluated cystometric and electromyographic parameters. Crucially, due to the robustness of the biological changes captured, all 12 previously identified significant parameters successfully maintained their statistical significance (p < 0.05) after the FDR adjustment. These updates have been transparently integrated into the Statistical Analysis section (Lines 273–276) and the footnote of Table 3 (Lines 333–336).

11. Reviewer Comment: Some non-significant EMG parameters raise questions about sensitivity!

Response: We appreciate this observation. The non-significant outcomes in specific EUS-EMG parameters (Phasic RMS, Phasic AUC, and Phasic Duration) reflect genuine neurobiological behavior rather than a lack of hardware sensitivity Discussion (Lines 357–365). The acute acetic acid model induces detrusor hyperactivity via afferent pathway irritation while leaving the efferent neuromuscular integrity of the external urethral sphincter (EUS) intact as detailed in the Discussion (Lines 415–422) and visualized in Figures 4 and 5.

12. Reviewer Comment: The authors should justify the sample size, clarify whether this is a proof-of-concept validation study

Response: We have explicitly revised the manuscript to clarify that this work is a technical proof-of-concept and pilot validation study across the Abstract (Line 26), Introduction (Lines 86–90), Statistical Analysis section (Lines 276–279), and Discussion (Lines 444–449). Regarding the sample size, the use of N = 15 subjects is methodologically justified by our strictly paired experimental design, where each animal served as its own baseline control before and after the acute challenge. This framework eliminates inter-animal biological variability from the error variance, significantly increasing statistical efficiency and data precision while strictly adhering to the ethical "Reduction" principle of animal research guidelines.

13. Reviewer Comment: The electrical performance metrics are impressive; however, these are bench measurements, not in vivo. In vivo noise sources (movement, electrode variability) are not quantified. It would strengthen the manuscript to include the signal-to-noise ratio in vivo comparison of raw vs filtered signals failure rates or unusable recordings

Response: We entirely agree that quantifying in vivo noise and operational reliability significantly strengthens the technical validation of the platform under real experimental conditions. Following the reviewer's suggestion, we have updated the manuscript to include these metrics. These quantifications have been integrated into Table 2 (Lines 287) and explicitly detailed in the In vivo Measurements section (Lines 303–307).

14. Reviewer Comment: Urethane anesthesia is an appropriate choice for preserving reflex micturition, but the manuscript should acknowledge its known effects on central modulation and sphincter coordination. Potential influence on EMG–pressure coupling is not discussed. Also, as a terminal anesthetic, it limits applicability to chronic or awake models

Response: We agree with this methodological perspective. While urethane is the standard for acute urodynamics due to reflex preservation, it is well-documented to depress supraspinal central modulation, which can influence fine EUS coordination and EMG–pressure coupling. Furthermore, its terminal classification inherently constrains the direct translation of these acute data to awake or chronic setups. To address this transparently, we have expanded the limitations section in the Discussion (Lines 403-407), explicitly addressing these anesthetic implica

Attachment Submitted filename: Response to Reviewers.docx

# Peer review: Decision Letter 1

**DOI:** 10.1371/journal.pone.0356999.r004

**Authors:** Richard Lee

12 Aug 2026

<p>UROREC: A cystometry and electromyography recording system for analyzing urinary incontinence parameters in rat models

PONE-D-26-17624R1

Dear Dr. Martinez,

We’re pleased to inform you that your manuscript has been judged scientifically suitable for publication and will be formally accepted for publication once it meets all outstanding technical requirements.

Within one week, you’ll receive an e-mail detailing the required amendments. When these have been addressed, you’ll receive a formal acceptance letter and your manuscript will be scheduled for publication.

An invoice will be generated when your article is formally accepted. Please note, if your institution has a publishing partnership with PLOS and your article meets the relevant criteria, all or part of your publication costs will be covered. Please make sure your user information is up-to-date by logging into Editorial Manager at Editorial Manager® and clicking the ‘Update My Information' link at the top of the page. For questions related to billing, please contact billing support.

If your institution or institutions have a press office, please notify them about your upcoming paper to help maximize its impact. If they’ll be preparing press materials, please inform our press team as soon as possible -- no later than 48 hours after receiving the formal acceptance. Your manuscript will remain under strict press embargo until 2 pm Eastern Time on the date of publication. For more information, please contact onepress@plos.org.

Kind regards,

Richard Kao Lee, M.D.

Academic Editor

PLOS One

Additional Editor Comments (optional):

Reviewers' comments:

Reviewer's Responses to Questions

**Comments to the Author**

Reviewer #1: All comments have been addressed

Reviewer #2: All comments have been addressed

**********

2. Is the manuscript technically sound, and do the data support the conclusions?

Reviewer #1: Yes

Reviewer #2: Yes

**********

3. Has the statistical analysis been performed appropriately and rigorously?

Reviewer #1: Yes

Reviewer #2: Yes

**********

4. Have the authors made all data underlying the findings in their manuscript fully available?

The PLOS Data policy

Reviewer #1: Yes

Reviewer #2: Yes

**********

5. Is the manuscript presented in an intelligible fashion and written in standard English?

Reviewer #1: Yes

Reviewer #2: Yes

**********

Reviewer #1: The authors have appropriately addressed all of my comments and concerns. I have no further comments, and the manuscript is suitable for publication in its current form.

Reviewer #2: All comments have been extensively addressed and reviewed

The addition of the comparison table is very useful for the authors and for the claim you are making for UROREC

Moving all the bioinformatics information to supplementary was appropriate

**********

what does this mean?). If published, this will include your full peer review and any attached files.

If you choose “no”, your identity will remain anonymous but your review may still be made public.

**Do you want your identity to be public for this peer review?**  For information about this choice, including consent withdrawal, please see our Privacy Policy

Reviewer #1: **Yes:** ILAHA ISALI

Reviewer #2: No

**********

# Editor report: Acceptance letter

**DOI:** 10.1371/journal.pone.0356999.r005

**Authors:** Richard Lee

PONE-D-26-17624R1

PLOS One

Dear Dr. Martínez,

I'm pleased to inform you that your manuscript has been deemed suitable for publication in PLOS One. Congratulations! Your manuscript is now being handed over to our production team.

At this stage, our production department will prepare your paper for publication. This includes ensuring the following:

* All references, tables, and figures are properly cited

* All relevant supporting information is included in the manuscript submission,

* There are no issues that prevent the paper from being properly typeset

You will receive further instructions from the production team, including instructions on how to review your proof when it is ready. Please keep in mind that we are working through a large volume of accepted articles, so please give us a few days to review your paper and let you know the next and final steps.

Lastly, if your institution or institutions have a press office, please let them know about your upcoming paper now to help maximize its impact. If they'll be preparing press materials, please inform our press team within the next 48 hours. Your manuscript will remain under strict press embargo until 2 pm Eastern Time on the date of publication. For more information, please contact onepress@plos.org.

You will receive an invoice from PLOS for your publication fee after your manuscript has reached the completed accept phase. If you receive an email requesting payment before acceptance or for any other service, this may be a phishing scheme. Learn how to identify phishing emails and protect your accounts at https://explore.plos.org/phishing.

If we can help with anything else, please email us at customercare@plos.org.

Thank you for submitting your work to PLOS One and supporting open access.

Kind regards,

PLOS ONE Editorial Office Staff

on behalf of

Dr. Richard Kao Lee

Academic Editor

PLOS One
